# Changes in Cecal Microbiota and Mucosal Gene Expression Revealed New Aspects of Epizootic Rabbit Enteropathy

**DOI:** 10.1371/journal.pone.0105707

**Published:** 2014-08-22

**Authors:** Christine Bäuerl, M. Carmen Collado, Manuel Zúñiga, Enrique Blas, Gaspar Pérez Martínez

**Affiliations:** 1 Laboratorio de Bacterias Lácticas y Probióticos, Departamento de Biotecnología, Instituto de Agroquímica y Tecnología de Alimentos (IATA), Consejo Superior de Investigaciones Científicas (Spanish National Research Council) (CSIC), Valencia, Spain; 2 Animal Nutrition Research Group, Institute of Animal Science and Technology, Polytechnic University of Valencia (UPV), Valencia, Spain; INIAV, I.P.- National Institute of Agriculture and Veterinary Research, Portugal

## Abstract

Epizootic Rabbit Enteropathy (ERE) is a severe disease of unknown aetiology that mainly affects post-weaning animals. Its incidence can be prevented by antibiotic treatment suggesting that bacterial elements are crucial for the development of the disease. Microbial dynamics and host responses during the disease were studied. Cecal microbiota was characterized in three rabbit groups (ERE-affected, healthy and healthy pretreated with antibiotics), followed by transcriptional analysis of cytokines and mucins in the cecal mucosa and vermix by q-rtPCR. In healthy animals, cecal microbiota with or without antibiotic pretreatment was very similar and dominated by *Alistipes* and *Ruminococcus*. Proportions of both genera decreased in ERE rabbits whereas *Bacteroides*, *Akkermansia* and *Rikenella* increased, as well as *Clostridium*, γ-Proteobacteria and other opportunistic and pathogenic species. The ERE group displayed remarkable dysbiosis and reduced taxonomic diversity. Transcription rate of mucins and inflammatory cytokines was very high in ERE rabbits, except IL-2, and its analysis revealed the existence of two clearly different gene expression patterns corresponding to *Inflammatory* and (mucin) *Secretory Profiles*. Furthermore, these profiles were associated to different bacterial species, suggesting that they may correspond to different stages of the disease. Other data obtained in this work reinforced the notion that ERE morbidity and mortality is possibly caused by an overgrowth of different pathogens in the gut of animals whose immune defence mechanisms seem not to be adequately responding.

## Introduction

The 19^th^ century witnessed the emergence of animal fancy and rabbits (*Oryctolagus cuniculus*) began to be raised also as pets, and at present days they are among the most important laboratory models. Rabbits are hindgut fermenters, their cecum has a large size and, with the stomach, it has a very relevant role in the digestive function. Coprophagy (caecotrophy) is a very characteristic habit of this species that aids to complete the digestion of vegetable components, facilitates the assimilation of proteins and other nutrients synthesized by cecal bacteria and maintains gut bacterial populations. Intestinal health of domestic rabbits is quite delicate and any disruption of the digestive process results in gastrointestinal diseases, most frequently related to diet or stress [1]. A high incidence of digestive diseases of unknown aetiology in young mammals is often related to distortions in microbiota composition [2]. The contribution of the gastrointestinal tract microbiota towards mammalian host health and performance is now widely accepted. Previous studies on rabbit cecal microbiota performed using classical culture-based techniques [3] and recently, by molecular techniques [4], showed that bacterial species are mainly strict anaerobes with predominance of the phylum Firmicutes over Bacteroides-Prevotella.

Epizootic rabbit enteropathy (ERE) is a serious farm rabbit disease that reaches 30–95% mortality, independent of the breed, with a particular incidence in the post-weaning period [5]. The main symptoms are cecal impaction, abdominal distension with gas and fluid accumulation in stomach and intestines, lethargy, hunched posture, subnormal temperature and copious clear mucus diarrhoea sometimes preceded by constipation, for which it is also called Mucoid Enteropathy. In acute form, clinical signs precede death by 1–3 days, or 7–9 days in a longer term condition. The most relevant histological feature is a remarkable goblet cells hyperplasia of ileum, cecum, vermix, saculated colon and colon [6]. In practice, exhaustive hygienic measures are required to constrain this epizoosis and, in some cases, only sustained antibiotic medication prevents ERE mortality. A great effort has been devoted to study the aetiology of ERE and it is now accepted that bacterial elements are involved in the pathogenesis, since antibiotic treatments are usually effective in the prevention of ERE, and also because the cecal content filtrate of ERE rabbits inoculated to healthy animals can reproduce the disease [7]. However, other identified and unidentified factors related to the environment, husbandry conditions and duration of the weaning period certainly participate in the development of ERE [8]. Diet is important, as the disease has been reproduced providing feed with a reduced fibre content and ligation of the cecum has been shown to be an invasive, but effective form to reproduce the disease [6], [8]. Exhaustive bacteriological analyses carried out in the past could not resolve the microorganisms involved, although clostridial species and coliforms have been most frequently found in diseased rabbits [6], [8]–[10].

A very relevant feature of ERE is the abundant production of mucus throughout the small, but remarkably, in the large intestine and cecum [8]. Gastrointestinal mucins produced by goblet cells are the main structural components of the mucus layer that has a crucial lubricating and protective role. Mucins are synthesized as a first line of defence and accumulate differential effector and regulatory responses against a plethora of commensal and pathogen microorganisms. Their synthesis is induced by pro-inflammatory stimuli stemming from pathogen invasion or chemical aggression of the epithelia, as part of the innate defence system, but also microorganisms and viruses efficiently recognize mucins as binding targets [11]. ERE histopathological signs have also been described [7], [8], but further biochemical, immunological or molecular techniques have not been implemented, and therefore, a deeper perspective of the disease is missing.

In this study, qPCR and pyrosequencing of 16S rDNA amplicons were used to describe and compare the cecal microbiota in healthy rabbits and to analyse the impact of antibiotic treatment and ERE on the microbiota composition. In addition, gene expression of mucins and pro-inflammatory cytokines was also determined and correlations with variations in cecal microbiota were estimated. This study also revealed new bacterial groups significantly related to ERE and new insights on the pathology of this disease.

## Materials and Methods

### Animals, feeding and housing conditions

Animals used in the experiment were three-way crossbreed rabbits from maternal lines A and V and paternal line R, developed by the Polytechnic University of Valencia (Animal Breeding Unit), Spain. They were grown in an experimental farm that kept standard commercial handling conditions at the Polytechnic University of Valencia. Lactation lasted 30 days and animals were separated in groups of ten per cage in the fattening area. After weaning a group of rabbits (antibiotic treatment) was fed *ad libitum* with commercial medicated pelleted rabbit feed (Nanta, Nutreco, Spain) containing lyncomycin (29 ppm), spectinomycin (29 ppm), tiamutin (40 ppm) and neomycin (121 ppm). A second group of rabbits was fed with a batch of the same feed without antibiotics. In order to increase the incidence of ERE these rabbits were housed in a fattening section of the farm fed without antibiotics, batch after batch, during 4 months previous to the experiment. At the age of 40 days, ten healthy animals and ten animals suffering typical ERE symptoms, like apathy, yellowish perianal fecal stain (mucus), cecal impaction and watery sound in the gut were sampled at random and from different cages of the section without antibiotics. Also ten rabbits with antibiotic treatment were selected randomly in the farm. All rabbits were collected on the same day. Healthy or ERE animals (gas in the stomach and impaction of the cecum) that upon necropsy could not be unequivocally diagnosed were eliminated from the study (total of 2). The complete clean cecum was extracted after ligation at ileo-cecal valve and the complete organ, including the vermix, was immediately frozen at −80°C until use.

This study was carried out in strict accordance with the Spanish national rules (RD223/1988 and RD1201/2005) that protect animals used in experimentation and other scientific purposes. The protocol was approved by the Committee on the Ethics of the Polytechnic University of Valencia (UPV). Due to their small size, animals were euthanized by cervical dislocation and then dissected.

### Nucleic acids extraction from cecal content, cecal mucosa and vermix

The frozen cecum content was fragmented in pieces of approximately 5 g and quickly defrosted. Then, total DNA was extracted from 200–300 mg samples of cecal content using a Qiagen stool DNA extraction kit (QIAgen, Hilden, Germany) according to manufacturer's instructions with a previous disruption with a bead beating step.

RNA samples for q-RT-PCR were obtained as follows. Cecal mucosa circular fragments of 10–15 mm diameter and transversal vermix sections of 5 mm were carefully excised and immediately submerged in TRIzol Reagent (Invitrogen). The tissue was homogenized with a Polytron device and total RNA was isolated using the RNeasy Mini Kit (Qiagen) according to the manufacturers' instructions. Contaminating genomic DNA was digested using Deoxyribonuclease I (Sigma) and then, RNA quantity and quality was evaluated using the Agilent 2100 Bioanalyser (Agilent). Reverse transcription reactions were performed using the Transcriptor First Strand cDNA Synthesis Kit (Roche) according to the instructions of the manufacturer.

### Quantitative real-time PCR (qPCR) analysis

The qPCRs of specific bacterial groups were conducted as previously described [12]. qPCR amplification and detection was performed in a LightCycler 480 Real-Time PCR System (Roche). Each reaction mixture of 10 µl consisted of SYBR Green PCR Master Mix (Roche), 0.5 µl of each of the specific primers [12] at a concentration of 0.25 µM, and 1 µl of template DNA. We also analysed methanobacteria using *Methanobrevibacter* genus-specific primers (MET-105f and MET-386r) and *M. smithii nif*H gene specific primers (Mnif-342 and Mnif-363r) as described elsewhere [13]. A melting curve analysis was made after amplification to assess the specificity of the amplification reaction. The bacterial concentration in each sample was calculated by interpolation of the obtained C_t_ values to standard curves. These were created using serial 10-fold dilution of pure culture-specific DNA fragments of known size corresponding to 10 to 10^9^ number of fragment gene copies/ml.

### Tagged PCR amplification of bacterial 16S ribosomal genes for pyrosequencing

A barcoded primer set based on universal primers 27F (5′-GAGTTTGATCMTGGCTCAG-3′), and 518R (5′-WTTACCGCGGCTGCTGG-3′), were used to amplify a 500 bps of the 16S rRNA genes encompassing the V3 region. The PCR was carried out using a high-fidelity KAPA-HiFi polymerase (KappaBiosystems, US) with an annealing temperature of 52°C and 20 cycles to minimize PCR biases. Purified PCR products were pooled in equimolar amounts, as 454 Roche protocols describe, and submitted for pyrosequencing using the Genome Sequencer FLX Titanium Series (454 Life Science, Branford, USA). All of the procedures followed the manufacturer's directions (454 Life Science) and were conducted at Macrogen (Seoul, South Korea). Both chains of all amplicons were sequenced to assure high quality data. Sequence data obtained in this work are available in MG-RAST (http://metagenomics.anl.gov/) as Rabbit-ERE project with accession Number 4543253.3.

### Gene expression analysis of rabbit genes through real time qPCR

For reverse transcription 5 µg of total RNA were used. The reactions were performed using the Transcriptor First Strand cDNA Synthesis Kit (Roche) according to the instructions of the manufacturer. Quantitative real-time PCR was performed on a LightCycler 480 Real Time PCR System using SYBR Green I Master Mix (Roche). PCR cycling conditions comprised an initial polymerase activation step at 95°C for 10 min followed by 40 cycles of 10 s at 95°C, 10 s at 60°C and 12 s at 72°C. qPCR primers were designed using the Primer-Blast tool (http://www.ncbi.nlm.nih.gov/) and are listed in Table S14. qPCR primers were validated to confirm efficiency through serial dilutions. PCR products were confirmed by agarose gel electrophoresis to yield a unique band and, additionally, after each qPCR run a dissociation curve was performed.

### Statistical analysis

For the analysis of qPCR data, IBM-SPSS 19.0 software (*SPSS* Inc., Chicago, IL, USA) was used. Due to non-normal distribution, microbial data are expressed as medians with interquartile ranges (IQR). Comparisons among data of more than two groups of rabbits were done by applying the Wilcoxon/Kruskal-Wallis test, and comparisons between data of two groups were done by applying the Mann-Whitney U test. The Bonferroni adjustment test was also applied to correct the significance of multiple test comparisons among three groups. The χ-square test was used to establish differences in the bacterial prevalence between the studied groups. A P<0.050 was considered statistically significant. The possible correlation between variables was studied by applying Pearson's correlation coefficient, and significance was established at 0.05%.Regarding pyrosequencing, low quality sequences were filtered out to remove sequences having a length shorter than 100 nucleotides from raw data sets. Sequences were aligned and classified against the SILVA comprehensive rRNA database (http://www.arb-silva.de/). A dereplicate request on the pipeline was used for identifying the representative sequences for each operational taxonomic unit (OTU) generated from the complete linkage clustering with a 97% similarity. After taxonomical assignment of pyrosequencing data, relative frequencies of different taxonomic categories obtained were calculated using the Statistical Analysis of Metagenomic Profiles program (STAMP v.2.0.0) [14]. Statistical differences between experimental rabbit groups were estimated by ANOVA analysis with the Games-Howell post-hoc test and the multiple test correction of Benjamini-Hochberg as implemented in STAMP. Rarefaction curves were calculated using the RarefactWin program (http://www.uga.edu/~strata/software/Software.html) and also, alpha diversity indexes were determined with the QIIME pipeline from rarefied tables using the Shannon-Wiener index for diversity, the Chao1 index for richness and also, Observed Species (number of unique OTUs) and Phylogenetic Distance (PD_whole). A beta diversity distance matrix was computed from the previously constructed OTU table using UniFrac analysis. Unweighted (presence/absence metrix) and weighted (presence/absence/abundance metrix) UniFrac distances were used to construct two- and three-dimensional Principal Coordinates Analyses (PCoA) plots. Biplots were generated as part of the beta diversity analysis in Qiime using genus level OTU tables showing principle coordinate clustering of samples alongside weighted taxonomic group data. Data on assigned sequences at the genus level shared between samples were used to generate a Venn diagram.

Relative gene-expression was quantified according to the efficiency-corrected method [15] using the REST 2009 software tool. Differences in input cDNA were normalized to glyceraldehyde-3-phosphate dehydrogenase (GAPDH) and β-actin (ACTB) expression.

Finally, correlation analysis was performed with the Statgraphics programme, as well as the Principal Component Analysis (PCA) of the sequences and expression data, with reciprocal projections, which were then drawn with SigmaPlot 10.0.

## Results

### Bacterial populations in the cecum of healthy rabbits

A total of 89,091 16S rRNA double stranded sequence reads were obtained from 10 cecal samples of healthy individuals fed with non-medicated feed. The average read length was 492.5 bp. Using the SILVA database for taxonomic assignment, the most common phyla were Firmicutes were Firmicutes (78.25% of total reads), Bacteroidetes (15.75%), Verrucomicrobia (2.40%), and Tenericutes (2.39%). Within Firmicutes, in the order Clostridiales, the most abundant families were Ruminococcaceae (42.48% of total reads) and Lachnospiraceae (34.85%) while in the order Bacteroidales, Rikenellaceae (6.38%) was the most abundant family. We found 90 different genera in cecal samples of healthy rabbits from which the predominant ones were *Alistipes* (5.63% of total reads), *Ruminococcus* (4.02%), *Akkermansia* (2.40%) and *Subdoligranulum* (2.28%). However, the 20 most abundant sequences (37.76% of total reads) could not be classified at the species level: 15 of them belonged to the class Clostridia (families Lachnospiraceae and Ruminococcaceae), including the most abundant sequence in healthy rabbits EF445173 already reported [3] (8.34% of total reads) (family Lachnospiraceae); 4 to the class Bacteroidia (family Rikenellaceae); and one belonged to the class Verrucomicrobia (genus *Akkermansia*; Tables S1, S2, S3, S4).

### Impact of antibiotic treatment and ERE on the cecal microbiota

Further 20 cecal samples of rabbits belonging to two additional groups, healthy treated with antibiotics (A) and ERE rabbits (E), were collected in order to be compared to the previous healthy rabbits without antibiotic treatment, used as control group (C). One sample failed in the antibiotic group (A) so that a total of 19 samples were analysed. After quality filtering and length trimming 199,217 16S rDNA reads were analysed, with an average number of taxonomically assigned high-quality double stranded reads of 10,485 per rabbit with an average size of 492 bp (Tables S1, S2, S3, S4).

The dominating Phyla in all rabbits were Firmicutes followed by Bacteroidetes. Class Clostridia (Firmicutes) was more abundant in healthy groups (A = 85.49%, C = 78.06%, E = 54.07%; corrected p<0.001) and class Bacteroidia in the ERE group (A = 8.83%, C = 15.73%, E = 22.18%; corrected p = 0.013). ERE rabbits showed a remarkable increase in Proteobacteria, particularly class γ-Proteobacteria (A = 0.016%, C = 0.023%, E = 10.03%; corrected p = 0.003). The number of Verrucomicrobiae was also higher in ERE (A = 0.84%, C = 2.40% E = 8.40%, corrected p = 0.009) mostly due to a great increase of reads of the genus *Akkermansia* (see below). Other taxons with lower total numbers showed remarkably different proportions in the ERE group: class Bacilli was more abundant in the ERE group (A = 0.07%, C = 0.02%, E = 1.1%), and Tenericutes (class Mollicutes; A = 3.68%, C = 2.39%, E = 0.44%) less frequent in diseased animals.

**Figure 1:**
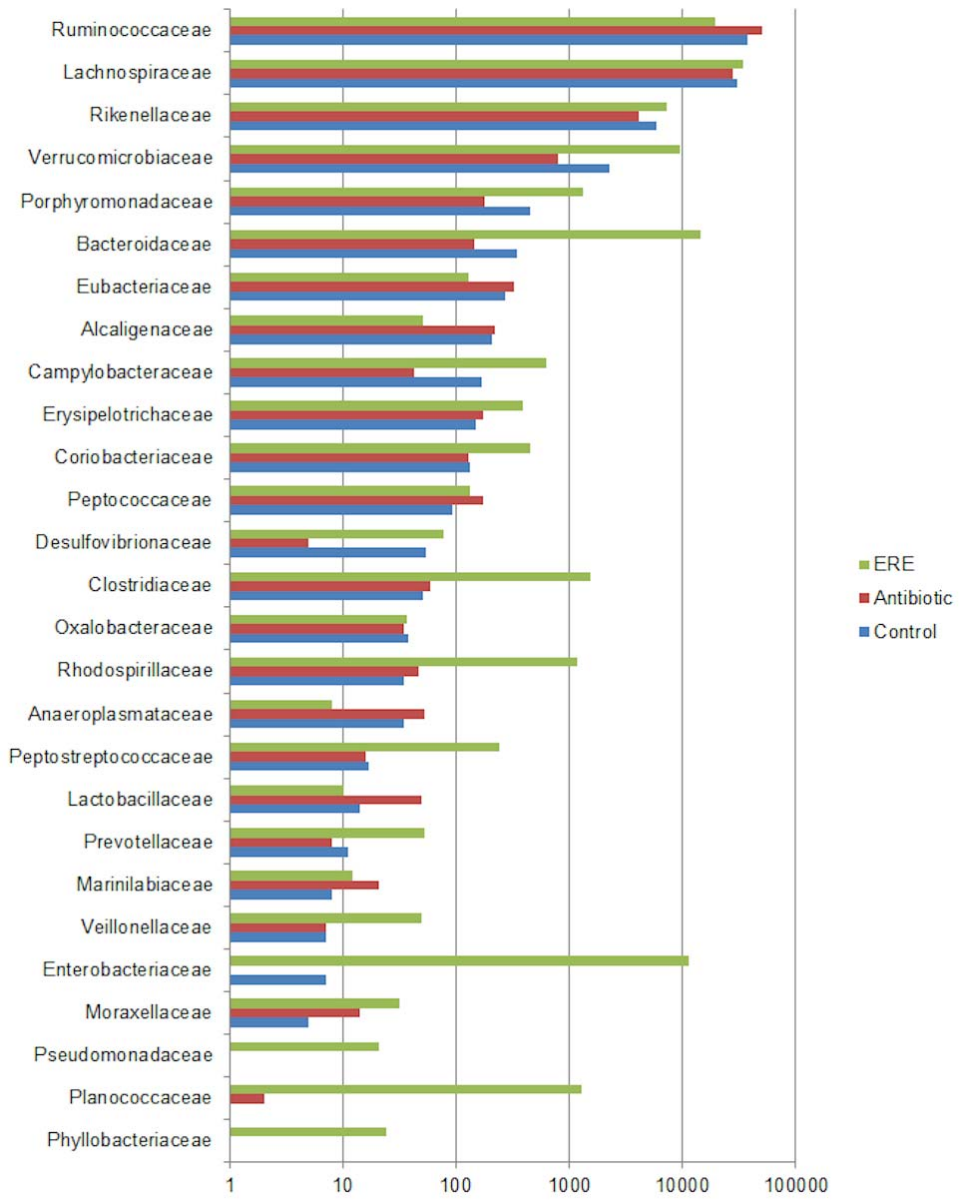
Total reads of bacterial families identified by SILVA after pyrosequencing of the 16S rRNA of the cecal content of rabbits. The graph represents the average for each of the three groups: control (white), antibiotic treatment (grey) and ERE (black).

**Figure 2:**
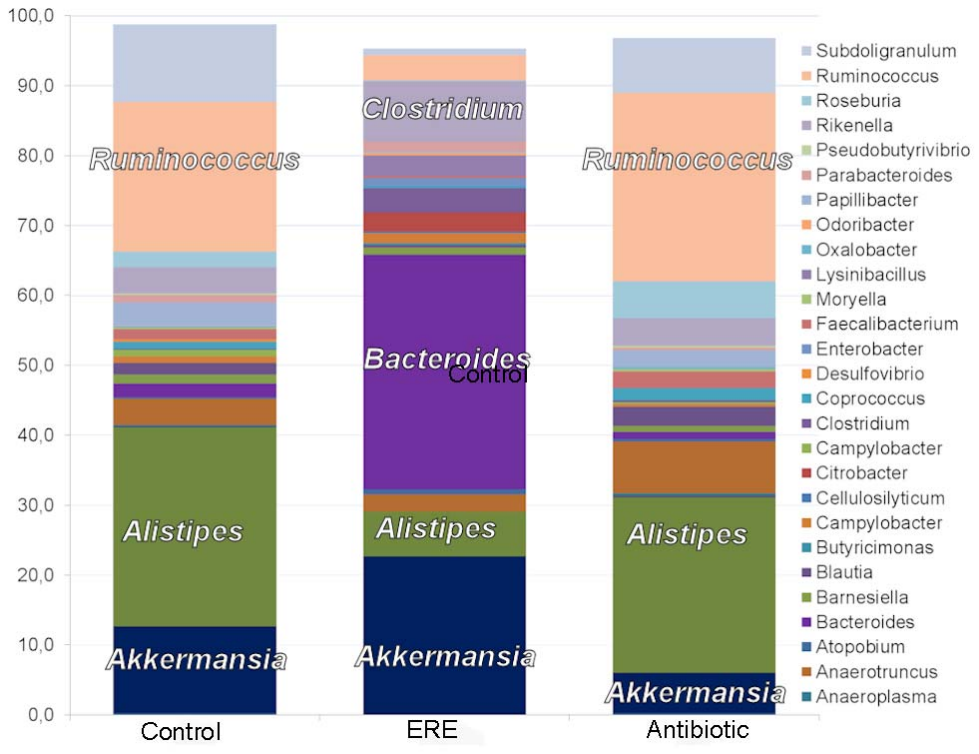
Proportional piled up column graph representing the average abundance of the different bacterial genera for the control group (healthy, no antibotics) (C), rabbits treated with antibiotics (A) and ERE animals (E). Most abundant genera are marked on the bar.

After the scrutiny of total counts of bacterial families in the three groups important differences were detected (Figure 1). Antibiotic treatment (A) reduced the number of reads in the families Clostridiaceae and Ruminococcaceae. A number of bacterial families were in high numbers or specifically present only in the ERE group, such as Verrucomicrobiaceae, Enterobacteriaceae and Bacteroidaceae (Figure 1). At a lower taxonomical level, the most frequent genera in healthy groups (A,C) were *Ruminococcus* and *Alistipes*, and in ERE rabbits *Bacteroides* (12,45%), *Akkermansia* (8,40%), *Escherichia* (8,25%), *Rikenella* (3.40%) as well as *Clostridium* (1.24%) showed a major presence (Figure 2, Table S4). The comparison of unclassified sequences showed that two Lachnospiraceae reads of unknown genus already reported obese humans DQ799912.1.1389 [16] and anaerobic sludge CU919535.1.1344 [17] accounted for 8.96% and 4.87% respectively of total identified sequences in ERE rabbits. In addition, potential toxin producing Firmicutes have also been found, such as species of the genus *Lysinibacillus*, with 0.99% of total reads in ERE cecal samples.

**Figure 3:**
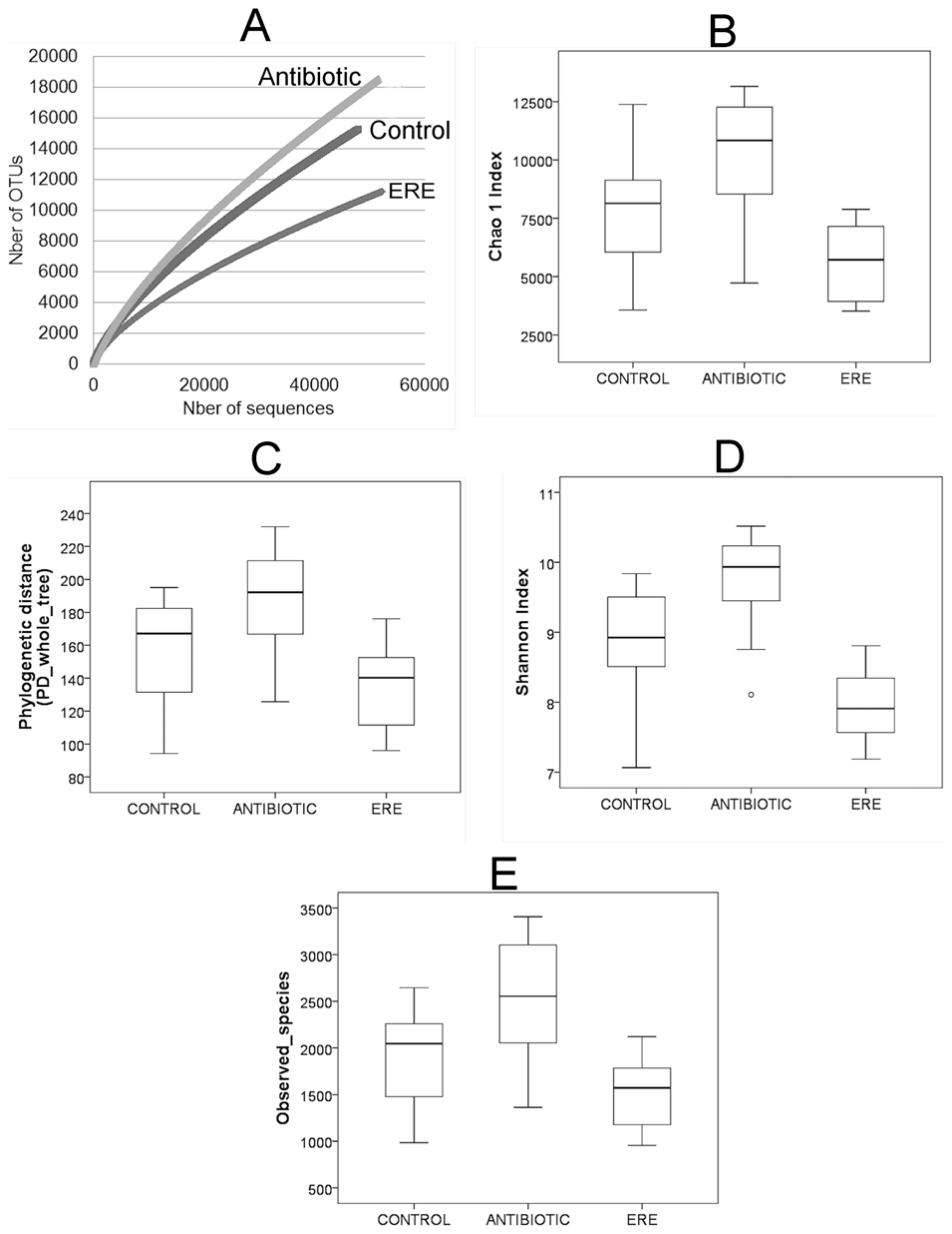
Rarefaction curves represent an approximation to the number of species identified for the total number of sequences obtained (A). The number of species was represented by the number of operational taxonomic units (OTUs). Curves were calculated with RarefactWin software at 97% similarity level corresponding to species-level phylotypes. Rarefaction analysis also included the estimation of the diversity of bacterial taxa in each sample with different metrics within the QIIME pipeline, such as the alpha diversity indices: Chao1 (B), Shannon (D), Phylogenetic distances (C) and Observed Species (E).

**Figure 4:**
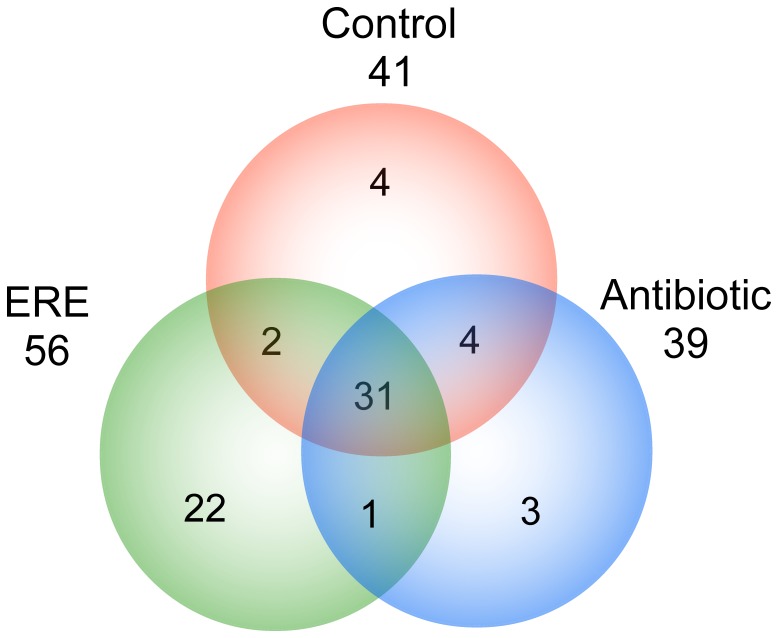
Venn diagram showing the relative abundance at the genus level based on the classification of the partial 16S rDNA bacterial sequences from cecal samples of antibiotic, healthy control and ERE groups using the RDP classifier software for taxonomy assignment: control group (healthy, no antibiotics) (Control), rabbits treated with antibiotics (Antibiotic) and ERE animals (ERE).

The three groups showed a remarkable diversity, with rarefaction curves indicating a number of Operational Taxonomic Units (OTUs) over 10,000 when reads were clustered at 97% sequence identity (the consensus value for determining species boundaries). Rarefaction curves, at OTU level, show that the ERE group had lower bacterial diversity than the control and antibiotic groups (Figure 3). The slope at the end of the curves of Antibiotic and Control groups suggests that microbiota is very diverse and a new efforts would be needed to represent the complete rabbit cecal microbiome. The 16S sequences of all individuals in each group were pooled, with an operational taxonomic-unit definition set at 97% sequence identity. An OTU was a cluster of 16S rRNA sequences which were over 95% identical, a conservative estimate for the boundary between species, established at 97% for full-length 16S gene sequences (Each rarefaction curve is plotted, along with its 95% confidence interval). As was seen in the primary analysis, the overall diversity in ERE group was significantly lower than that in control subjects and antibiotic treatment. Overall diversity of taxa within the samples was determined by alpha diversity metrics (Chao1 metric estimates the species richness; Shannon estimates the diversity; the Observed Species; Phylogenetic Distance (PD_whole_tree)) showed lower diversity, phylogenetic richness and evenness in ERE group compared to those observed in the other two groups: control and antibiotic group (Figure 3). Venn diagram shows a broader perspective, where Control and Antibiotic groups share most of the bacterial families and genera. A core of 13 families and 24 genera were present in ERE, antibiotic and control group (Figure 4).

**Figure 5:**
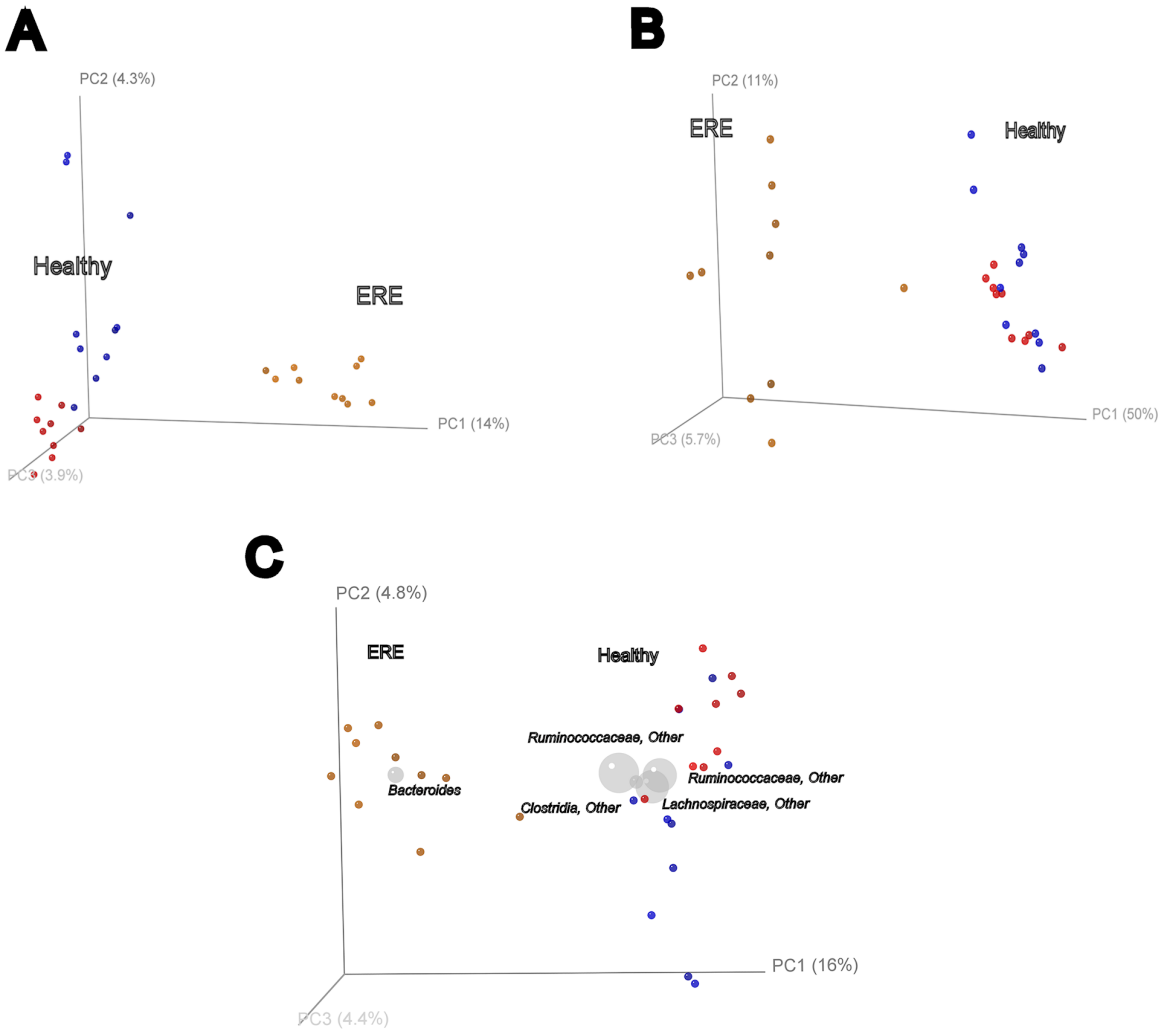
Principal Coordinates Analysis (PCoA) 3D plots generated with UniFrac showing clustering of OTUs bacterial groups of cecal samples from ERE, and Healthy (Control and Antibiotic) groups. PCoA is a Multidimensional Scaling Method that allows to explore and to visualize dissimilarities of phylogenetic data from a distance matrix imported from RDP, assigning a location to each sample in a 3D graphics. Panel A, unweighted plot; panel B, weighted plot (taking into account the relative abundance of each taxon in the samples); panel C, Biplot representation of PCoA of unweighted, pairwise Unifrac distances showing clustering of bacterial groups cecal samples from ERE, Control and Antibiotic groups. Blue spheres are Control rabbits, red spheres are the Antibiotic group and light brown spheres are the ERE rabbits. In panel C, the Biplot shows the projection of taxa positions (grey spheres) that represent weighted averages of the coordinates of all samples. The size of the spheres is proportional to the relative abundance of the taxon.

Principal Coodinates Analysis (PCoA) of the samples clearly separated the ERE rabbits from the rest of group samples in both, weighted (Figure 5, panel A) and unweighed (Figure 5, panel B) plots (PC1 accounting for 14% of the variance in unweight and 50% in weight Unifrac analysis), indicating that the ERE microbiota is compositionally distinct. Healthy individuals with or without antibiotic treatment (Antibiotic,Control) clustered together and separately from the ERE group of animals. This indicated that microbiota from groups *Antibiotic* and *Control* are not significantly different, but a remarkable dysbiosis is affecting ERE rabbits. In order to explore taxonomic driving factors of patterns in a PCoA bacterial genera were plotted in the same diagram of the unweighted PCoA of all samples (Biplot), where relative abundance of these taxons were also represented by the sphere sizes. The genus *Bacteroides* clearly clustered with ERE samples, clustering of healthy samples was driven by two uncharacterized genera of the Ruminococcaceae family, one genus of Lachnospiraceae and a less abundant genus of the Clostridia class (Figure 5, panel C).

Finally, qPCR was performed to analyse the presence of certain species and to confirm significant differences in the proportion of specific taxonomic groups between ERE, antibiotic and healthy control group samples (Table S5). Significantly higher levels of *Akkermansia muciniphila*, *Bacteroides-Prevotella* and *Clostridium coccoides group* were observed in ERE groups compared to those observed in control (p = 0.001, p = 0.042 and p = 0.050, respectively) and antibiotic (p = 0.002, p = 0.002 and p = 0.049, respectively). Furthermore, we determined the presence of bacteria from the genus *Methanobrevibacter* as example of Archaea in rabbit cecal samples. A significantly higher level of the bacteria of the genus *Methanobrevibacter* was observed in the ERE group compared to the healthy control and antibiotic groups (p<0.0001), although the species *M. smithii* was detected more often in healthy controls (4/10, 40%) and antibiotic group (3/10, 30%) than in ERE group (1/10, 10%). However, overall proportions of metanobacteria (*Methanobrevibacter*) are very small, as amplified molecules of 16S rDNA were two (C group) to five (E group) orders of magnitude below total bacteria.

### Mucin and cytokine expression in the cecum

**Figure 6:**
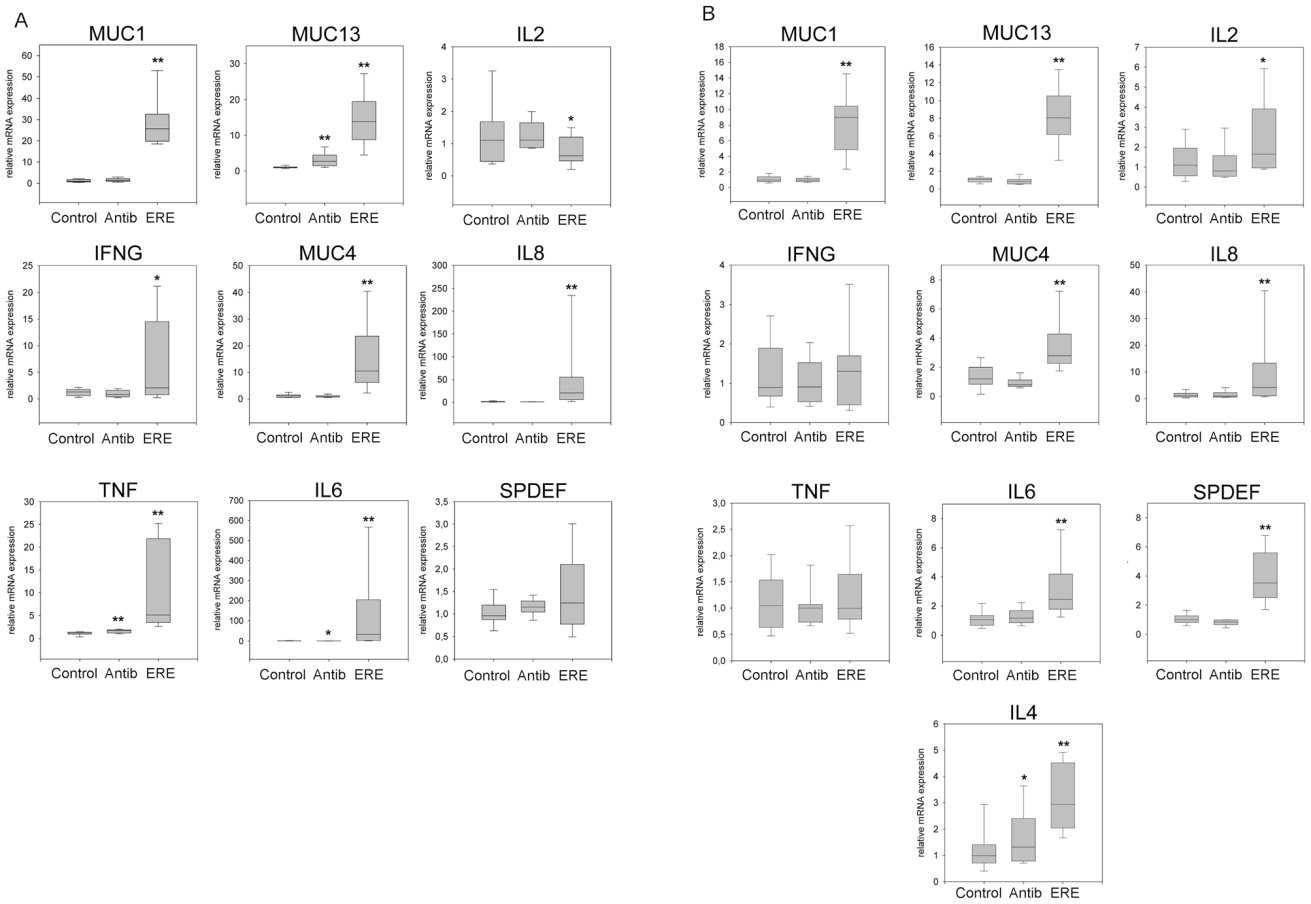
Graphs representing relative gene expression data determined by q-RT-PCR. Values are calculated relative to the Control group and normalized to endogenous ACTB and GAPDH expression. Box plots show the median value (solid line inside boxes), the limits of box represent the 25th and 75th percentile, and whiskers depict the 5th and 95th percentile (*P<0.05 vs. Control; **P<0.001 vs. Control).

One of Epizootic Rabbit Enteropathy main physiological symptoms is the abundant secretion of mucins at the cecum and colon [18], for which monitoring their synthesis during the course of the disease, compared to that in healthy animals, was considered a priority issue. Mucin secretion by the goblet cells is known to be induced by inflammatory signals, frequently after pathogen challenges [11], hence we searched in the rabbit genome project (http://www.ncbi.nlm.nih.gov/genome?term=oryctolagus%20cuniculus) in order to design suitable primers for q-RT-PCR of mucin and cytokine encoding genes. MUC1, MUC4 and MUC13 were successfully tested and also the SAM-pointed domain-containing Ets-like factor gene (SPDEF), an activator of goblet cells which is induced by pro-inflammatory signals and by pathogen stimuli, that also regulates mucine expression [19]. Homologous gene to MUC2, the dominant intestinal mucin in humans, was not available at the onset of this study. Also primers for rabbit pro-inflammatory cytokines and lymphocyte differentiation precursors were designed (TNF-α, IL-4, IL-6, IL-8, IFN-γ and IL-2). Gene expression of all the genes was determined in cecal mucosa samples of all 30 rabbits (Table S7), and expression data could be successfully calculated with the exception of IL-4 whose C_t_ was too high to allow reliable calculations in cecal mucosa. Transcription data were calculated as relative expression of ERE (E) and antibiotic groups (A) against the mean value of the healthy control (C). Rabbits suffering ERE displayed an increased transcription level of all mucins, especially MUC1 (Figure 6, panel A). The average expression values of SPDEF had no significant increase in any of the three groups, however, particular differences in the ERE group will be dissected below. Expression of cytokines IL-8, TNF-α and IL-6 were also significantly upregulated (Figure 6, panel A). This result showed a direct linkage between the disease status and mucin synthesis in the cecal epithelium of ERE rabbits, indicating the presence of a strong pro-inflammatory stimulus in the cecal contents. IFN-γ showed a small induction, whereas IL-2 had a moderate decrease in transcription. However, a great variability was noticed in the transcription rates of the cytokines within the ERE group, for this reason we decided to further analyse this group (see below). Little differences were found between the transcription rates of rabbits treated with antibiotics (A) and the control group (C), besides a slight tendency to overproduce mucins in the A group (Figure 6, panel A). Furthermore, gene expression data in cecal mucosae of ERE was analysed in detail. A remarkable and highly significant correlation was detected between TNF-α and IL-6 and IL-8, as expected from their direct connection with canonical pro-inflammatory signaling pathways (i.e. NF-κB), and also connection of IFN-γ with TNF-α and IL-6 and IL-8 could be confirmed. Regarding mucin expression, we found a positive correlation (low significance, p≤0.1) of SPDEF expression with MUC13 suggesting regulatory connections, and a negative correlation with IL-2 and INFγ, suggesting a reverse (repressing) regulatory connection, but no relationship of SPDEF with the two other mucins MUC1 and MUC4. However, MUC13 negative correlation to INFγ and TNF-α was greater and more significant, and also that of MUC1 with IL-2 (Table S6).

**Figure 7:**
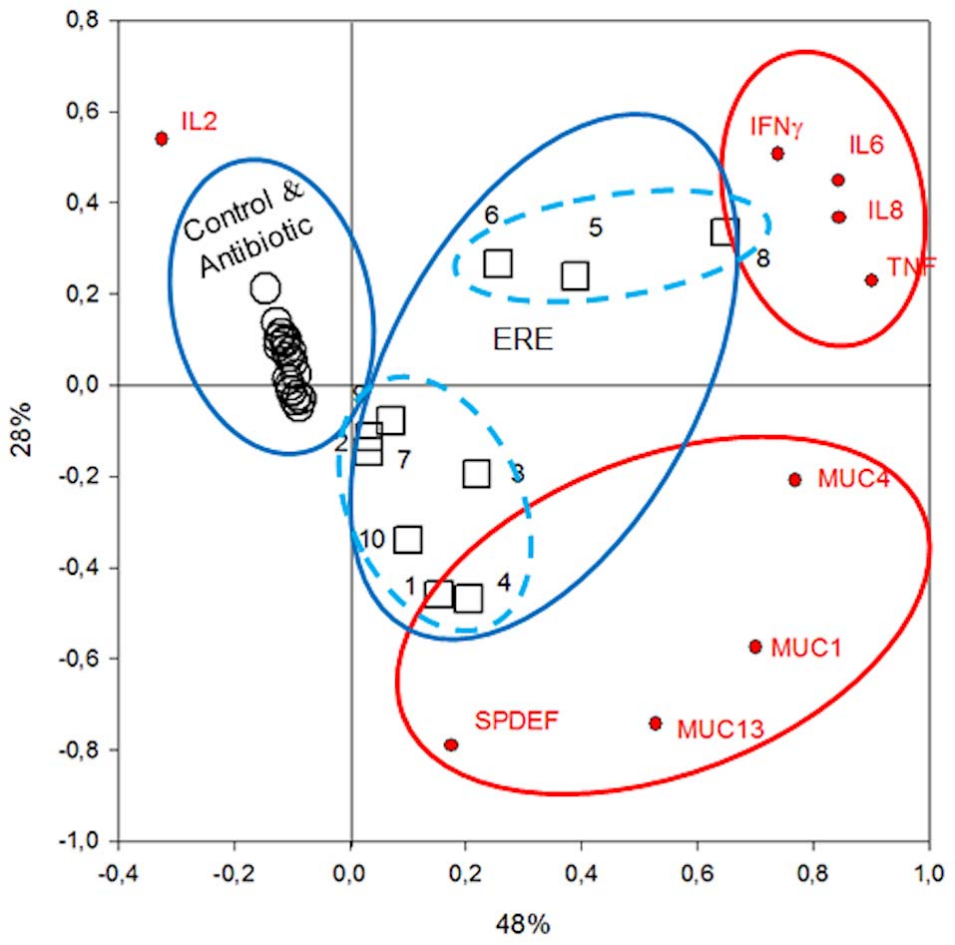
PCA of cytokine gene expression, where taxonomic data from each rabbit sample are projected. Red circles indicate groupings of gene expression data. Blue circles indicate the position of individual rabbits.

Then, PCA of gene expression data in the cecal mucosa was performed and drawn (Figure 7). Variables clearly grouped in two clusters, one including cytokines (IFN-γ, TNF-α, IL-6 and IL-8) and the second, mucins (MUC13, MUC1, MUC4 and the regulator SPDEF). They were separated along the ordinate axis (second component) and IL-2 remained distant from either group, underlining an independent expression pattern. In fact, expression of this cytokine showed very small variation between healthy and ERE rabbits. When data from all rabbits were projected on the gene expression PCA, healthy individuals (Control and Antibiotic groups) clustered tightly at the negative (left) side of the abcise axis (PC1), while ERE animals were all projected in the positives values (right). Interestingly, rabbits suffering the disease were separated in two subsets along the ordinate axis (PC2) with rabbits E5, E6, E8 (n = 3) and E1, E2, E3, E4, E7, E9, E10 (n = 7) closer to the cluster of inflammatory cytokines and mucins, respectively. In fact the expression profiles of cytokines and mucins of these two subgroups were very different. Samples of the first group (E5, E6, E8) had very high expression of pro-inflammatory cytokines, IL-6x16, IL-8x8, TNF-αx3 and IFN-γx11 fold greater than the other group, while samples in the second group (E1, E2, E3, E4, E7, E9, E10) had higher expression of mucins (Table 1, Table S8), for which they were assigned to *Inflammatory Profile* and *Secretory Profile*, respectively. Unfortunately the *n* of the *Inflammatory Profile* was low (n = 3) for further comparisons.

**Table 1:** Mean values and standard deviation of gene expression values, relative to control rabbits, of the two likely profiles (Inflammatory and Secretory) found in rabbits suffering ERE.

	Cecum					Vermix				
	Inflammatory profile (n = 3)		Secretory profile (n = 7)			Inflammatory profile (n = 3)		Secretory profile (n = 7)		
	Mean	SDev	Mean	SDev	P value	Mean	SDev	Mean	SDev	P value
**MUC1**	21.62	4.07	31.75**	11.66	0.039	6.84	1.95	8.80	4.46	0.180
**MUC13**	7.04	2.60	17.35**	5.96	0.003	7.90	2.55	8.47	3.39	0.390
**IL2**	1.07	0.48	0.62	0.38	0.120	3.88	2.56	1.81	1.10	0.146
**IFN-γ**	17.24**	4.02	1.54	1.14	0.009	1.84	1.65	1.11	0.66	0.263
**MUC4**	22.15	17.57	12.62	10.52	0.227	4.89	2.30	2.71	1.06	0.119
**IL8**	123.20	113.26	15.63	16.93	0.120	22.35	19.25	3.95	3.88	0.119
**TNF-α**	20.81**	6.06	6.47	6.72	0.013	1.88*	0.87	0.94	0.32	0.097
**IL6**	341.28*	229.66	21.24	27.43	0.068	4.74	2.57	2.45	1.12	0.130
**IL4**	-	-	-	-	-	4.17**	0.88	2.74	1.08	0.040
**SPDEF**	0.74	0.32	1.71**	0.79	0.013	3.47	1.76	4.29	1.82	0.269

Asterisks represent averages significantly greater in one profile than the other, with significance:

(*)p≤0.1;

(**)p≤0.05.

### Mucin and cytokine expression in the vermix

Transcription was also assayed with vermix samples, as these samples could be enriched in lymphocytes (Figure 6, panel B). Interestingly, there were significant differences between transcription levels of Control samples and ERE in all genes studied, except IFN-γ and TNF-α, and mucin genes (MUC1, MUC13, MUC4) and cytokine genes IL-4, IL-6, IL-8 followed a similar tendency to that in the cecal mucosa, although IL-4 could not be quantified in the cecal mucosa. Induction of mucins mRNA was much lower than in the cecum (Figure 6, panel A). No significant differences were found between antibiotic and the control groups (Figure 6, panel B).

When comparing only gene expression of vermix and cecal samples of ERE animals, we detected no upregulation of IFN-γ and TNF-α in vermix, but a moderate overexpression of IL-2 (two fold) and SPDEF (four fold). Expression data from vermix within the ERE group were further analysed in order to ascertain if there could also be two profiles, as those found in the cecum samples. Interestingly, IL-4 expression in vermix samples could be quantified and it was significantly greater in the *Inflammatory Profile* than in the *Secretory Profile*.

### Cecal microbiota and gene expression relationships

Different statistical analysis were run to determine likely correlations between individual gene expression data and bacterial frequencies, with the aim to relate possible organisms or bacterial taxons to the pathogenesis of ERE. As expected, global correlation analysis of relative gene expression and microbial groups in all rabbits showed that high expression of MUC1, MUC13, MUC4, IL-8 and IL-6 and TNF-α was in all cases significantly bound to high reads of those bacterial taxons most abundant in ERE, such as orders Verrucomicrobiales, Enterobacteriales and Bacteroidales, and on the contrary, high expression of those markers were correlated to low levels of Clostridiales reads belonging to the class Clostridia (families Lachnospiraceae and Ruminococcaceae), described as associated to healthy rabbit samples (see above). Also at lower taxon level (genus), with the exception of *Alistipes* and *Anaerotruncus*, all most abundant genera were significantly correlated to high or low mucins and pro-inflammatory cytokines expression, depending whether they were most abundant in the ERE or Healthy groups, respectively (Tables S9, S10, S11, S12). Also we tried to find, through correlation analysis, organisms significantly bound to the gene expression *Profiles* identified (*Inflammatory* and *Secretory*) within the ERE group, but significant information could not be obtained due to the low number of rabbits in the *Inflammatory Profile*. However, plain PCA at species (organisms) level performed with most abundant sequences in the ERE group, with projection of gene expression data, indicated that uncultured and uncharacterized isolates of the genus *Akkermansia*, *Alistipes* and *Bacteriodes* clustered with IL-6, IL-8, TNFα and IFNγ and in the proximity of rabbit samples 5, 6 and 8 (*Inflammatory Profile*) (Figure S1). Diverse sequences were positioned next to MUC1 and MUC13 in the PCA plot with representatives of *Akkermansia*, *Escherichia* (four sequences), *Bacteroides* (two sequences), *Clostridium* and three unidentified species and genus of the family *Lachnospiraceae*. However, the four sequences of *Escherichia* and one *Lachnospiraceae* were closer to the core of MUC1 and MUC13 projection. This stresses the differences between the *Inflammatory and Secretory Profiles* and suggests they may be bound to specific microbial environment/elements.

## Discussion

Studies using molecular methods for the analysis of the gut microbiota in rabbits are not abundant. Capillary electrophoresis single-stranded conformation polymorphism (CE-SSCP) and denaturing gradient gel electrophoresis (DGGE) were useful to determine the similarity between bacterial populations of the cecal content and soft faeces [20] on diets with different fibre content [21] and to draw a general quantitative map of the evolution of the microbiota along the rabbit's life [4]. Cloning of 16S RNA genes in *Escherichia coli* offered a more precise identification of the bacterial inhabitants of rabbit's gut, however, the procedure introduced qualitative and quantitative biases. In a first inventory, Abecia et al. (2005) [21] showed the relevance in rabbit's cecal content of Clostridia (Lachnospiraceae, Ruminococcaceae) and Verrucomicrobia, and reported a number of new unidentified sequences. Authors described the absence of sequences of the *Prevotella*–*Bacteroides* group, however, nowadays identification databases have been enriched and we could classify all those sequences (Group 5, in Abecia et al, 2005), in the Bacteroidetes phylum (Table S13). In a later work, a larger number of sequences (228) were analysed and distributed in 70 OTUs in nine clusters, where the majority of sequences belonged to the phylum *Firmicutes* (94%), only three were *Bacteroidetes* (4%) and one *Verrucomicrobia* [3]. Pyrosequencing has been recently used to investigate the microbial composition of a variety of cecal filtrates and fractions that reproduced ERE in rabbits, but no statistical differences were found between samples in that work [22]. Our work showed that cecal microbiota in healthy rabbits is dominated by Firmicutes (78.25% of total OTUs), Bacteroidetes (15.75%), Verrucomirobia (2.40%) and Tenericutes (2.39%) and this quantitatively similar to that found in other monogastric herbivores (hindgut fermenters) [23] and in mouse, as the closest model, in which also Firmicutes dominate over Bacteroidetes [24], [25].

In this work, we obtained 289,000 good quality sequences (both strands) from 29 samples to facilitate the comparative analysis of healthy rabbits treated or not with antibiotics and rabbits suffering ERE. The first surprising result was that there were no significant differences due to the antibiotic treatment, despite that it was reported that antibiotics reduced the microbial diversity in rabbits [22], [26], as they did in mice [27] and humans [28]. The fact that we found no significant differences between control rabbits and the antibiotic group might be due to the prolonged preventive medication in the experimental farm, a situation that could generate a uniform environmental population installed in the farm that ultimately colonize rabbits. However, this uniform microbiological environment could facilitate the detection of bacterial pathogens involved in ERE, or at least to establish the microbiological scenario in the cecum of rabbits suffering ERE. The most relevant feature of ERE cecal microbiota is a remarkable dysbiosis and reduced taxonomic diversity. An extreme environment must be generated during early pathogenesis, as occurs in a number of other pathologies with a marked inflammatory profile, where particularly *γ-*Proteobacteria proliferate, such as human inflammatory bowel disease (IBD), necrotizing enterocolitis in human infants [29] or piglet short bowel syndrome and murine model of Crohn's disease [30]–[33]. Quantitative and qualitative differences between healthy and ERE rabbits have been found, such as the abundance of *Verrucomicrobiae*, particularly the genus *Akkermansia*. *A. muciniphila* is a typical intestinal anaerobe frequently associated to human healthy mucosa [34]. Its high prevalence in ERE cecal samples could be related to their mucin scavenger character [35], [36], however, *A. muciniphila* can aggravate gut inflammation when there is prevalent a *Salmonella* infection [37]. *Ruminococcus* is the most relevant genus of the *Firmicutes* phylum dominant in healthy individuals, that notably decreases in ERE, despite the fact that this is generally an efficient mucin degrading genus [30], [31]. Another component of the healthy microbiota is the genus *Alistipes*. Both play an important role in the degradation of vegetable feed components and the production of short chain fatty acids [38], [39], hence controlling Bacteroidetes numbers [40], and therefore, could constitute essential components of rabbit cecal microbiota and candidates as probiotics. Methanobacteria (Archaea) were reported in low numbers in the cecal content of rabbits, only present after day 7 of life [4]. Our data obtained by qPCR confirmed it. Production of methane has been shown to be inversely proportional to the size of animals. Carbon excreted as methane is represents a metabolic loss, hence in the case of small herbivores like rabbits, this may represent a degree of fermentative specialization surpassing the efficiency of ruminants and other herbivores of greater body mass [41].

ERE rabbits exhibited a decrease of Clostridia and increase of class Bacteroidia and γ-Proteobacteria, but also Verrucomicrobia due to the increase of the genus *Akkermansia*. The genus *Lysinibacillus* (3% in ERE) and other microorganisms not typically pathogenic like *Eggerthella sinensis*, *Bacteroides thetaiotaomicron* were significantly present in ERE and not the healthy groups (C,A). They are indicators of fecal contamination as they are normally found in other mammals intestine, like dogs and humans [42], [43]. Of note, the cecal content of ERE rabbits contains a great diversity of genera, like *Lysinibacillus*, *Escherichia*, *Clostridium* and *Bacteroides*, that include potential pathogenic species. Although some are innocuous gut colonizers, species belonging to the genus *Lysinibacillus* have been described to produce tetrodotoxin, the lethal neurotoxin typical of puffer fish, normally produced by *Vibrio alginolyticus* [44], [45]. Also isolates of *Lysinibacillus fusiformis* produce citotoxins and are causative agents of necrotizing ulcerative gingivitis and painful tooth infections [46]. Among coliforms, 16S sequences of potential pathogens have been identified in ERE rabbits, such as *Escherichia coli* E24377A, an enterotoxigenic *E. coli* (ETEC) [47] , or other clinical isolates, as *E. coli* UMN026 and *E. coli* IAI39. Hence, a number of opportunistic pathogens and fecal species typically isolated in other animals, but unusual or absent in healthy rabbits, have also been found in ERE samples that could not be specifically associated with the origin of the disease, but which notably contribute to the dysbiosis and certainly will produce no benefit to rabbits welfare and health. Since the lack of fiber in feed [8] and cecum ligation are direct forms to reproduce ERE [6], it could be inferred that either an infective agent, a physiological or even a fortuitous event occurs in some young rabbits that collapses intestinal motility and illeo-cecal valve, and prevents normal cecal evacuation leading to undesirable bacteria overgrowth in the cecum. In such case, we propose that the true cause of ERE should be searched at earlier stages, in still-healthy animals. The causative agent of ERE has been associated in the past with species of the genus *Clostridium* and *Escherichia* [18], [48]. In addition to those, it could be interesting to isolate bacterial species with unidentified sequences to test their infective potential, such as various strains of the genus *Bacteroides*, or two strains of the family Lachnospiraceae (DQ799912.1.1389 and CU919535.1.1344) that accounted for 13,8% of total identified sequences in ERE rabbits.

No experimental records have been found describing gene expression in intestinal samples of rabbits. Gene expression analysis through q-RT-PCR of 9 different markers highlighted the extreme pro-inflammatory scenario of cecal mucosa in ERE rabbits. Since mucin secretion constitutes a typical feature of the disease, upregulation of mucins gene expression (MUC1, MUC4, MUC13) was expected in the cecal mucosa of ERE rabbits. Gastrointestinal mucins play a critical role in innate host defences, they are the main structural components of the mucus layer and are produced by goblet cells [11]. We also analysed SAM-pointed domain ETS factor (SPDEF), which is a regulator induced by pro-inflammatory signals and pathogens that promotes differentiation of goblet cells in different organs, including the intestine, and is required for activation of goblet cell associated gene expression such as mucins [19]. Its expression level in ERE cecal mucosae was only moderately upregulated and only direct correlation with MUC13 expression could be found.

A number of possible pathogens found in ERE samples could provide pathogen associated molecular patterns (PAMPs) to interact with pattern recognition receptors. As known, Gram positive bacteria peptidoglycans can be recognized by the surface receptor TLR2 or intracellularly by NOD1 and NOD2 leading to an increased production of pro-inflammatory cytokines, such as TNF-α, IL-6 and IL-8 [49], as in the case of the recognition of *Clostridium difficile* [50]. Flagellins and lipopolysaccharides of the outer membrane of coliforms are also powerful inflammatory signals through TLR5 and CD14/TLR4 consortium, respectively, inducing NF-κB inflammatory cascade [43], [51]. In fact, Gram negative bacteria seem to be more efficient inducing IL-6 and IL-8 than Gram positives [52]. Typically during an infective process, the innate immune system is mobilized within the first few days in order to control infection, while adaptive immune responses that involve generation of immunological memory and expansion of receptors with relevant specificities are observed 4 to 7 days after infection [53]. We found that ERE cecal samples had a very significant upregulation of markers of the canonical NF-κB pro-inflammatory pathway (TNF-α. IL-6 and IL-8), as it would correspond to PAMP stimuli. This high expression of inflammatory markers and mucins in affected animals showed two patterns that clearly grouped samples with very high induction of TNF-α, IL-6 and IL-8 and moderate expression of mucins (*Inflammatory Profile*) and those with very high expression of mucins and moderately high expression of cytokines (*Secretory Profile*). The expression pattern of IFN-γ of the two profiles suggests that Th1 differentiation may be initiated in the *Inflammatory Profile*, while, lower transcription of IFN-γ and TNF-α and higher transcription of IL-2, IL-4 and IL-6 in the vermix of animals with *Inflammatory Profile* also suggest that they may be starting an adaptive immune response. Certainly, these two profiles have differential features, including different bacterial species associated, hence, it could be suggested that these patterns may correspond to different types or stages of the disease. Studies are in progress to relate these profiles to clinical symptoms.

As in human gastrointestinal inflammatory diseases, dysbiosis and reduced diversity are general features also found in ERE [29], [54], and qualitative similarities can be found with bacterial populations in human inflammatory processes [55], [56]. However, dysbiosis may not be the cause but rather a consequence of the disease.

In summary, this work has produced an extensive characterization of healthy rabbits' cecal microbiota and in ERE affected animals and for the first time, gene expression of mucins and proinflammatory cytoquines has been analysed in rabbits. Firmicutes dominated in the cecum of healthy rabbits followed by Bacteroidetes, a general pattern found in hindgut herbivores. Healthy microbiota showed high proportions of *Ruminococcus* and uncultured species of the Lachnospiraceae family. Samples of ERE were rich opportunistic and pathogen bacteria, including *A. muciniphila*, all of them contributing to aggravate the disease. Candidate bacterial causative agents previously described have been found, like *Clostridium* or *Escherichia* species, but also uncultured species the genus *Bacteroides* and particularly two Lachnospiraceae strains, have been significantly related to the disease. High expression level of mucins and pro-inflammatory cytokines was detected in ERE samples as possible response to a powerful bacterial pattern stimulation, but low T helper cell differentiation could be inferred. Then, the combination of gene expression with microbiota data analysis led to the description of two disease profiles (*Inflammatory* and *Secretory*), suggesting different types or stages of the disease. Taking into account the limited previous knowledge on rabbit ERE microbiota, comparison of microbiota from ERE and healthy rabbits with such depth was absolutely required. In order to disclose the causes of this disease, and considering that it hits predominantly post-weaning rabbits and the existence of two distinct profiles, future research should include a longitudinal study of the caecotrophs' microbiota and immune parameters in young rabbits along this period, putative doe's protecting factors, epithelial and immune maturation promoters of which young rabbits are deprived at weaning.

## Supporting Information

Figure S1 **PCA of organisms (species) level found in ERE, with projection of gene expression data and the individuals.** PCA was performed with sequences whose reads were >100 and which were 10 fold greater in ERE than average of Healthy rabbits, groups Antibiotic and Control.
(TIF)

Table S1 (A) Total of identified reads obtained through bar coded pyrosequencing of 16S rDNA identified by SILVA database. (B) Number of reads assigned per phylum by the Silva database after pysequencing.
(DOC)

Table S2 **List of the main bacterial Classes characterized through pyrosequencing of 16S rDNA of the cecal content in rabbits with ERE (Enteropathy), healthy (Control) or healthy with antibiotics (Antibiotics).**
(XLS)

Table S3 **List of the main bacterial Families characterized through pyrosequencing of 16S rDNA of the cecal content in rabbits with ERE (Enteropathy), healthy (Control) or healthy with antibiotics (Antibiotics).**
(XLS)

Table S4 **List of the main bacterial Genera characterized through pyrosequencing of 16S rDNA of the cecal content in rabbits with ERE (Enteropathy), healthy (Control) or healthy with antibiotics (Antibiotics).**
(XLS)

Table S5 **Microbiota composition in rabbit cecal content analyzed by qPCR.** Data are shown as prevalence (Pr), median, and interquartile range (IQR). Statistical analysis was calculated using the Kruskal-Wallis test.
(DOC)

Table S6 **Pearson's correlation of cecal mucosa gene expression data in all rabbits.**
(DOC)

Table S7 **Gene expression profiles in all rabbits expressed as Fold Change relative to the average values of the ten rabbits in the Control group.**
(DOCX)

Table S8 **Gene expression in** ***Secretory and Inflammatory Profiles*** **in the cecal mucosa of ERE rabbits expressed as Fold Change relative to the average values of the ten rabbits in the Control group.**
(DOCX)

Table S9 **Correlation coefficient in the all rabbits between gene expression data (Ct relative to control) and OTUs.** Full taxonomic affiliation of OTUs is shown to get a broader perspective. Data for OTUs with a total frequency at least of 0.1%.
(DOCX)

Table S10 **Correlation coefficient in the ERE group: Orders Correlation Indices found in ERE rabbits between gene expression data (Ct relative to control) and OTUs.** Full taxonomic affiliation of OTUs is shown to get a broader perspective. Data for OTUs with a total frequency at least of 0.1%.
(DOCX)

Table S11 **Correlation coefficients of the frequency of OTUs (frequency >0.1%) and gene expression in the ERE group.**
(DOCX)

Table S12 **Correlation coefficients in the ERE group of frequency of reads per Genera with gene expression (C_t_ relative to control) (counts from SILVA and correl coef Pearson and bilateral significance levels).** Correlation coefficient in the ERE group: Orders Correlation Indices found in ERE rabbits between gene expression data (Ct) and OTUs. Data with a total frequency at least of 0.1%.
(DOCX)

Table S13 **Identification of sequences from Group 5 described in Abecia et al. 2005** [21] **.**
(DOCX)

Table S14 **List of oligonucleotides used in this study for the quantification of gene expression through q-RT-PCR in rabbit's cecal mucosa**.
(DOCX)
