# Supplementary figures and images for: Changes in Cecal Microbiota and Mucosal Gene Expression Revealed New Aspects of Epizootic Rabbit Enteropathy
Source: PLoS One. 2014 Aug 22;9(8):e105707. doi: 10.1371/journal.pone.0105707 (PMC4141808; doi:10.1371/journal.pone.0105707)

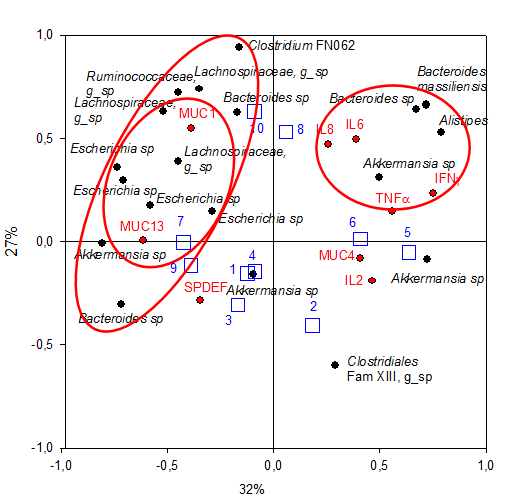

Supplement: Figure S1 — PCA of organisms (species) level found in ERE, with projection of gene expression data and the individuals. PCA was performed with sequences whose reads were >100 and which were 10 fold greater in ERE than average of Healthy rabbits, groups Antibiotic and Control. (TIF) [file pone.0105707.s001.tif]
