# Supplementary material for: Changes in Cecal Microbiota and Mucosal Gene Expression Revealed New Aspects of Epizootic Rabbit Enteropathy
Source: PLoS One. 2014 Aug 22;9(8):e105707. doi: 10.1371/journal.pone.0105707 (PMC4141808; doi:10.1371/journal.pone.0105707)
Supplement: Table S1 — (A) Total of identified reads obtained through bar coded pyrosequencing of 16S rDNA identified by SILVA database. (B) Number of reads assigned per phylum by the Silva database after pysequencing. (DOC) [file pone.0105707.s002.doc]

**SUPPLEMENTARY INFORMATION**

**Table S1 (A)**.- Total identified reads obtained through bar coded pyrosequencing of 16S rDNA identified by the SILVA database

| **Rabbit samples** | **Total bacteria** | **Class** | **Order** | **Family** | **Genus** | **Organism** |
| --- | --- | --- | --- | --- | --- | --- |
| Antibiotic | 92.016 | 91.654 | 88.532 | 85.083 | 13.765 | 4.023 |
| Control | 89.091 | 88.549 | 86.652 | 78.122 | 18.055 | 3.758 |
| Enteropathy | 107.201 | 106.111 | 105.818 | 103.243 | 42.370 | 3.907 |
| TOTAL | 288.308 | 286.314 | 281.002 | 266.448 | 74.190 | 11.688 |

**Table S1 (B)**.- Number of reads assigned per phylum by the Silva database after pysequencing

| **Phylum** | **Antibiotic** | **Control** | **Entheropathy** |
| --- | --- | --- | --- |
| Actinobacteria | 141 | 141 | 463 |
| Acidobacteria | - | 1 | - |
| Bacteroidetes | 7773 | 15042 | 24503 |
| Cyanobacteria | 154 | 273 | 319 |
| Fibrobacteria | 2 | - | - |
| Firmicutes | 79369 | 68662 | 58005 |
| Fusobacteria | 13 | 10 | 8 |
| Gemmatimonadetes | 2 | - | - |
| Lentisphaerae | 5 | 5 | 1 |
| Proteobacteria | 400 | 536 | 13640 |
| Spirochaetes | 3 | - | 3 |
| Synergistetes | 9 | 6 | 1 |
| Tenericutes | 3323 | 2076 | 386 |
| Verrucomicrobia | 791 | 2239 | 9611 |
| TOTAL | 91985 | 88991 | 106940 |
