# Supplementary material for: Changes in Cecal Microbiota and Mucosal Gene Expression Revealed New Aspects of Epizootic Rabbit Enteropathy
Source: PLoS One. 2014 Aug 22;9(8):e105707. doi: 10.1371/journal.pone.0105707 (PMC4141808; doi:10.1371/journal.pone.0105707)
Supplement: Table S5 — Microbiota composition in rabbit cecal content analyzed by qPCR. Data are shown as prevalence (Pr), median, and interquartile range (IQR). Statistical analysis was calculated using the Kruskal-Wallis test. (DOC) [file pone.0105707.s006.doc]

**SUPPLEMENTARY INFORMATION**

**Table S5 *.-*** Microbiota composition in rabbit cecal content analysed by qPCR. Data are shown as prevalence (Pr), median, and interquartile range (IQR). Statistical analysis was calculated using the Kruskal-Wallis test

| **Caecal samples** | **ERE (n=10)** | | | **Antibiotic(n=10)** | | | **Healthy Control(n=10)** | | | **P-value** |
| --- | --- | --- | --- | --- | --- | --- | --- | --- | --- | --- |
|  | Pr^1^ | **Median** | **IQR^2^** | Pr^1^ | **Median** | **IQR** | Pr^1^ | **Median** | **IQR** |  |
| Total Bacteria | 10 | 8.25 | 8.10-8.40 | 10 | 8.83 | 8.65-8.91 | 10 | 9.30 | 8.94-9.30 | 0.001 |
| *Akkermansia muciniphila* | 10 | 8.04 | 7.27-8.33 | 10 | 6.60 | 5.58-7.40 | 10 | 6.60 | 6.31-7.07 | 0.003* |
| *Bacteroides* group | 10 | 8.00 | 7.64-8.11 | 10 | 7.55 | 7.48-7.59 | 10 | 7.55 | 7.23-7.92 | 0.013* |
| *Clostridium coccoides* | 10 | 6.98 | 6.65-7.53 | 10 | 6.73 | 6.40-6.84 | 10 | 6.55 | 5.76-7.04 | 0.077 |
| *Clostridium leptum* | 10 | 7.10 | 6.68-7.21 | 10 | 7.32 | 7.06-7.44 | 9 | **6.79** | 6.50-6.97 | 0.080 |
| *Methanobrevibacter genus* | 10 | **6.47** | 5.71-6.77 | 10 | 5.61 | 5.36-6.23 | 10 | 4.32 | 3.25-4.68 | 0.0001* |
| *Methanobrevibacter smithii* | 1 | - | - | 3 | 2.57 | - | 4 | 2.43 | - | - |
| *Metanobrevibacter vs total Bacteria* |  | 0,016595 |  |  | 0,000602 |  |  | 1,04x10^-5^ |  |  |

1) Prevalence (Pr) reflects the percentage of positive amplifications from total samples analysed by PCR (n=number of samples analysed)

*) Statistical analysis was calculated using the Kruskal-Wallis test. Statistical differences were corrected for a multiple comparison test using the Bonferroni adjustment, and significant differences among the groups were considered as having a P-value < 0.017(0.05/3)

-) statistical analysis was not possible
