# Supplementary material for: Changes in Cecal Microbiota and Mucosal Gene Expression Revealed New Aspects of Epizootic Rabbit Enteropathy
Source: PLoS One. 2014 Aug 22;9(8):e105707. doi: 10.1371/journal.pone.0105707 (PMC4141808; doi:10.1371/journal.pone.0105707)
Supplement: Table S6 — Pearson's correlation of cecal mucosa gene expression data in all rabbits. (DOC) [file pone.0105707.s007.doc]

**SUPPLEMENTARY INFORMATION**

**Table S6.-** Pearson’s correlation of cecal mucosa gene expression data in all rabbits.

|  | MUC13 | IL2 | IFN-γ | MUC4 | IL8 | TNF-α | IL6 | SPDEF |
| --- | --- | --- | --- | --- | --- | --- | --- | --- |
| MUC1 | 0,627* | **-0,636**** | -0,512 | -0,132 | -0,245 | -0,361 | -0,314 | 0,380 |
| MUC13 |  | -0,445 | **-0,708**** | 0,004 | -0,494 | **-0,762***** | -0,551* | 0,581* |
| IL2 |  |  | 0,475 | 0,305 | 0,044 | 0,105 | 0,187 | -0,597* |
| IFN-γ |  |  |  | 0,186 | 0,591* | **0,678**** | **0,746**** | -0,558* |
| MUC4 |  |  |  |  | 0,230 | 0,370 | 0,211 | 0,137 |
| IL8 |  |  |  |  |  | **0,770***** | **0,971***** | -0,258 |
| TNF-α |  |  |  |  |  |  | **0,751**** | -0,249 |
| IL6 |  |  |  |  |  |  |  | -0,393 |

*) p≤ 0.1

**) p≤ 0.05

***) p≤ 0.01
