# Supplementary material for: Changes in Cecal Microbiota and Mucosal Gene Expression Revealed New Aspects of Epizootic Rabbit Enteropathy
Source: PLoS One. 2014 Aug 22;9(8):e105707. doi: 10.1371/journal.pone.0105707 (PMC4141808; doi:10.1371/journal.pone.0105707)
Supplement: Table S7 — Gene expression profiles in all rabbits expressed as Fold Change relative to the average values of the ten rabbits in the Control group. (DOCX) [file pone.0105707.s008.docx]

**TABLE S7**.- Gene expression profiles in all rabbits expressed as Fold Change relative to the average values of the ten rabbits in the Control group

| **Gene expression profiles in Healthy Control rabbits** | | | | | | | | | | |
| --- | --- | --- | --- | --- | --- | --- | --- | --- | --- | --- |
|  | **C1** | **C2** | **C3** | **C4** | **C5** | **C6** | **C7** | **C8** | **C9** | **C10** |
| **MUC1** | 0,72 | 0,71 | 0,662 | 1,415 | 1,066 | 2,116 | 0,332 | 1,603 | 2,109 | 0,823 |
| **MUC13** | 0,946 | 0,682 | 0,772 | 0,949 | 0,894 | 1,56 | 0,919 | 0,995 | 1,617 | 1,026 |
| **IL2** | 1,409 | 0,808 | 1,973 | 0,434 | 1,582 | 0,36 | 3,39 | 0,758 | 0,452 | 1,547 |
| **IFNG** | 0,681 | 1,696 | 1,611 | 1,055 | 2,009 | 0,254 | 2,203 | 0,655 | 0,448 | 1,547 |
| **MUC4** | 0,515 | 0,675 | 1,14 | 0,568 | 2,246 | 0,485 | 2,462 | 1,15 | 1,249 | 1,153 |
| **IL8** | 1,008 | 0,965 | 2,264 | 0,733 | 1,56 | 0,363 | 3,657 | 0,991 | 0,244 | 1,236 |
| **TNF** | 1,337 | 1,137 | 1,35 | 1,205 | 1,192 | 0,369 | 1,339 | 1,09 | 0,435 | 1,45 |
| **IL6** | 0,729 | 0,492 | 2,187 | 0,827 | 1,183 | 0,836 | 2,854 | 1,329 | 0,449 | 0,916 |
| **SPDEF** | 0,929 | 0,9 | 1,149 | 1,571 | 0,953 | 1,112 | 0,965 | 0,606 | 1,337 | 0,799 |
| **Gene expression profiles in ERE rabbits** | | | | |  |  |  |  |  |  |
|  | **E1** | **E2** | **E3** | **E4** | **E5** | **E6** | **E7** | **E8** | **E9** | **E10** |
| **MUC1** | 30,857 | 18,913 | 30,205 | 37,411 | 18,502 | 20,123 | 25,147 | 26,222 | 25,001 | 54,71 |
| **MUC13** | 20,03 | 12,006 | 9,778 | 27,986 | 4,11 | 7,924 | 16,709 | 9,085 | 15,684 | 19,249 |
| **IL2** | 0,575 | 0,746 | 0,183 | 0,521 | 1,511 | 1,15 | 0,667 | 0,558 | 1,351 | 0,304 |
| **IFNG** | 1,953 | 3,573 | 2,19 | 0,711 | 14,013 | 21,741 | 0,826 | 15,954 | 1,337 | 0,172 |
| **MUC4** | 20,706 | 2,03 | 11,348 | 32,224 | 41,379 | 6,933 | 8,442 | 18,146 | 9,581 | 4,042 |
| **IL8** | 9,508 | 8,636 | 46,901 | 5,84 | 79,744 | 38,112 | 5,841 | 251,756 | 31,536 | 1,134 |
| **TNF** | 4,633 | 5,583 | 21,542 | 4,15 | 22,961 | 13,967 | 2,503 | 25,495 | 3,486 | 3,365 |
| **IL6** | 4,378 | 23,088 | 41,184 | 2,308 | 216,146 | 201,356 | 3,069 | 606,333 | 73,544 | 1,094 |
| **SPDEF** | 3,103 | 1,45 | 2,08 | 2,17 | 0,654 | 0,477 | 0,811 | 1,103 | 0,995 | 1,381 |
| **Gene expression profiles in Healthy rabbits treated with Antibiotics** | | | | | | | | |  |  |
|  | **A1** | **A2** | **A3** | **A4** | **A5** | **A6** | **A7** | **A8** | **A9** | **A10** |
| **MUC1** | 2,973 | 1,676 | 0,557 | 1,964 | 1,505 | 1,208 | 0,883 | 0,931 | 2,868 | 0,97 |
| **MUC13** | 6,851 | 5,905 | 0,969 | 2,852 | 1,558 | 2,543 | 3,115 | 1,092 | 2,538 | 3,99 |
| **IL2** | 0,883 | 0,861 | 2,008 | 0,986 | 0,884 | 1,904 | 1,234 | 0,855 | 1,565 | 1,288 |
| **IFNG** | 0,586 | 0,359 | 1,427 | 0,919 | 0,702 | 1,96 | 1,578 | 0,213 | 1,674 | 0,418 |
| **MUC4** | 0,585 | 0,754 | 0,79 | 1,809 | 0,859 | 1,022 | 0,813 | 0,823 | 1,698 | 0,478 |
| **IL8** | 0,725 | 0,745 | 1,347 | 1,024 | 0,791 | 1,1 | 1,365 | 0,626 | 1,153 | 0,966 |
| **TNF** | 1,345 | 1,142 | 1,824 | 2,014 | 0,961 | 1,83 | 1,869 | 1,75 | 1,646 | 1,185 |
| **IL6** | 0,891 | 0,821 | 0,737 | 0,074 | 0,677 | 0,607 | 1,063 | 0,543 | 1,089 | 0,671 |
| **SPDEF** | 1,203 | 1,195 | 0,839 | 1,106 | 1,323 | 1,084 | 1,051 | 1,431 | 1,011 | 1,274 |
