# Supplementary material for: Changes in Cecal Microbiota and Mucosal Gene Expression Revealed New Aspects of Epizootic Rabbit Enteropathy
Source: PLoS One. 2014 Aug 22;9(8):e105707. doi: 10.1371/journal.pone.0105707 (PMC4141808; doi:10.1371/journal.pone.0105707)
Supplement: Table S8 — Gene expression in Secretory and Inflammatory Profiles in the cecal mucosa of ERE rabbits expressed as Fold Change relative to the average values of the ten rabbits in the Control group. (DOCX) [file pone.0105707.s009.docx]

**TABLE S8**.- Gene expression in Secretory and Inflammatory profiles in the cecal mucosa of ERE rabbits, expressed as Fold Change relative to the average values of the ten rabbits in the Control group

| **SECRETORY PROFILE** | | |  |  |  |  |  |  |  | | |  | | |
| --- | --- | --- | --- | --- | --- | --- | --- | --- | --- | --- | --- | --- | --- | --- |
|  |  |  |  |  |  |  |  |  |  | | |  | | |
| **Rabbit** | **E1** | **E2** | **E3** | **E4** | **E7** | **E9** | **E10** | **MEAN** | | **ST DEV** | | |  |  |
| **MUC1** | 30,857 | 18,913 | 30,205 | 37,411 | 25,147 | 25,001 | 54,71 | **31,75** | | 11,66 | | |  |  |
| **MUC13** | 20,03 | 12,006 | 9,778 | 27,986 | 16,709 | 15,684 | 19,249 | **17,35** | | 5,96 | | |  |  |
| **IL2** | 0,575 | 0,746 | 0,183 | 0,521 | 0,667 | 1,351 | 0,304 | **0,62** | | 0,38 | | |  |  |
| **IFNG** | 1,953 | 3,573 | 2,19 | 0,711 | 0,826 | 1,337 | 0,172 | **1,54** | | 1,14 | | |  |  |
| **MUC4** | 20,706 | 2,03 | 11,348 | 32,224 | 8,442 | 9,581 | 4,042 | **12,62** | | 10,52 | | |  |  |
| **IL8** | 9,508 | 8,636 | 46,901 | 5,84 | 5,841 | 31,536 | 1,134 | **15,63** | | 16,93 | | |  |  |
| **TNF** | 4,633 | 5,583 | 21,542 | 4,15 | 2,503 | 3,486 | 3,365 | **6,47** | | 6,72 | | |  |  |
| **IL6** | 4,378 | 23,088 | 41,184 | 2,308 | 3,069 | 73,544 | 1,094 | **21,24** | | 27,43 | | |  |  |
| **SPDEF** | 3,103 | 1,45 | 2,08 | 2,17 | 0,811 | 0,995 | 1,381 | **1,71** | | 0,79 | | |  |  |
|  |  |  |  |  |  |  |  |  | |  | | |  |  |
| **INFLAMMATORY PROFILE** | | |  |  |  |  |  |  | |  | | |  |  |
|  |  |  |  |  |  |  |  |  | |  | | |  |  |
| **Rabbit** | **E5** | **E6** | **E8** |  |  |  |  | **MEAN** | | **ST DEV** | | |  |  |
| **MUC1** | 18,502 | 20,123 | 26,222 |  |  |  |  | **21,62** | | | 4,07 | | |  |
| **MUC13** | 4,11 | 7,924 | 9,085 |  |  |  |  | **7,04** | | | 2,60 | | |  |
| **IL2** | 1,511 | 1,15 | 0,558 |  |  |  |  | **1,07** | | | 0,48 | | |  |
| **IFNG** | 14,013 | 21,741 | 15,954 |  |  |  |  | **17,24** | | | 4,02 | | |  |
| **MUC4** | 41,379 | 6,933 | 18,146 |  |  |  |  | **22,15** | | | 17,57 | | |  |
| **IL8** | 79,744 | 38,112 | 251,756 |  |  |  |  | **123,20** | | | 113,26 | | |  |
| **TNF** | 22,961 | 13,967 | 25,495 |  |  |  |  | **20,81** | | | 6,06 | | |  |
| **IL6** | 216,146 | 201,356 | 606,333 |  |  |  |  | **341,28** | | | 229,66 | | |  |
| **SPDEF** | 0,654 | 0,477 | 1,103 |  |  |  |  | **0,74** | | | 0,32 | | |  |
|  |  |  |  |  |  |  |  |  |  | | |  | | |
