# Supplementary material for: Changes in Cecal Microbiota and Mucosal Gene Expression Revealed New Aspects of Epizootic Rabbit Enteropathy
Source: PLoS One. 2014 Aug 22;9(8):e105707. doi: 10.1371/journal.pone.0105707 (PMC4141808; doi:10.1371/journal.pone.0105707)
Supplement: Table S9 — Correlation coefficient in the all rabbits between gene expression data (Ct relative to control) and OTUs. Full taxonomic affiliation of OTUs is shown to get a broader perspective. Data for OTUs with a total frequency at least of 0.1%. (DOCX) [file pone.0105707.s010.docx]

**Table S9**.- Correlation coefficient in the all rabbits between gene expression data (Ct relative to control) and OTUs at Order level. Full taxonomic affiliation of OTUs is shown to get a broader perspective. Data for OTUs with a total frequency at least of 0.1%

| **PHYLUM** | **CLASS** | **ORDER** | Frec % | **MUC1** | **MUC13** | **IL-2** | **IFNγ** | **MUC4** | **IL-8** | **TNFα** | **IL-6** | **SPDEF** |
| --- | --- | --- | --- | --- | --- | --- | --- | --- | --- | --- | --- | --- |
| Actinobacteria | Actinobacteria | Coriobacteriales | 0.29 | 0.348 | **0.418*** | -0.011 | **0.402*** | **0.585*** | **0.663*** | **0.597*** | **0.715*** | 0.033 |
| Bacteroidetes | Bacteroidia | Bacteroidales | 11.11 | **0.443*** | 0.289 | -0.333 | **0.378*** | **0.506*** | **0.475*** | **0.474*** | 0.462 | -0.042 |
| Firmicutes | Bacilli | Bacillales | 0.35 | **0.773*** | **0.720*** | -0.334 | 0.282 | **0.771*** | **0.693*** | **0.746*** | **0.704*** | -0.001 |
| Firmicutes | Bacilli | Lactobacillales | 0.3 | 0.198 | 0.251 | -0.152 | **-0.385*** | -0.330 | -0.281 | -0.181 | -0.058 | 0.045 |
| Firmicutes | Clostridia | Clostridiales | 72.5 | **-0.496*** | **-0.361*** | 0.311 | **-0.368*** | **-0.567*** | **-0.546*** | **-0.506*** | **-0.568*** | 0.085 |
| Firmicutes | Erysipelotrichi | Erysipelotrichales | 0.25 | **0.761*** | **0.745*** | **-0.404*** | 0.164 | **0.642*** | **0.602*** | **0.619*** | **0.679*** | 0.058 |
| Proteobacteria | Alpha-Proteobacteria | RF32 | 0.2 | **0.659*** | **0.612*** | -0.290 | 0.317 | **0.733*** | **0.671*** | **0.663*** | **0.717*** | 0.012 |
| Proteobacteria | Epsilon-Proteobacteria | Campylobacterales | 0.26 | **0.438*** | 0.319 | **-0.370*** | 0.158 | **0.373*** | 0.277 | 0.359 | 0.285 | -0.068 |
| Proteobacteria | Gamma-Proteobacteria | Enterobacteriales | 3.24 | **0.804*** | **0.782*** | -0.356 | 0.322 | **0.773*** | **0.740*** | **0.784*** | **0.757*** | 0.046 |
| Verrucomicrobia | Verrucomicrobiae | Verrucomicrobiales | 3.89 | **0.591*** | **0.551*** | -0.290 | 0.180 | **0.657*** | **0.585*** | **0.507*** | **0.730*** | 0.060 |

*) p≥ 0.05
