# Supplementary material for: Changes in Cecal Microbiota and Mucosal Gene Expression Revealed New Aspects of Epizootic Rabbit Enteropathy
Source: PLoS One. 2014 Aug 22;9(8):e105707. doi: 10.1371/journal.pone.0105707 (PMC4141808; doi:10.1371/journal.pone.0105707)
Supplement: Table S10 — Correlation coefficient in the ERE group: Orders Correlation Indices found in ERE rabbits between gene expression data (Ct relative to control) and OTUs. Full taxonomic affiliation of OTUs is shown to get a broader perspective. Data for OTUs with a total frequency at least of 0.1%. (DOCX) [file pone.0105707.s011.docx]

**Table S10**.- Correlation coefficient in the ERE group: Orders Correlation Indices found in ERE rabbits between gene expression data (Ct relative to control) and OTUs. Full taxonomic affiliation of OTUs is shown to get a broader perspective. Data for OTUs with a total frequency at least of 0.1%

| **PHYLUM** | **CLASS** | **ORDER** | **Freq** | **MUC1** | **MUC13** | **IL2** | **IFNγ** | **MUC4** | **IL8** | **TNFα** | **IL6** | **SPDEF** |
| --- | --- | --- | --- | --- | --- | --- | --- | --- | --- | --- | --- | --- |
| Bacteroidetes | Bacteroidia | Bacteroidales | 17.30% | -0.285 | **-0.770**^*^ | 0.152 | 0.552 | 0.127 | 0.612 | 0.576 | **0.636*** | -**0.648*** |
| Cyanobacteria | 4C0d-2 | YS2 | 0.20% | **-0.673*** | -**0.636*** | 0.345 | **0.673*** | -0.188 | 0.309 | 0.442 | 0.370 | -0.455 |
| Firmicutes | Erysipelotrichi | Erysipelotrichales | 0.10% | 0.273 | 0.539 | -0.236 | -**0.661*** | -0.079 | -0.430 | -0.564 | -0.430 | 0.309 |
| Proteobacteria | Epsilonproteobacteria | Campylobacterales | 0.30% | -0.261 | -0.418 | 0.467 | 0.212 | 0.139 | 0.345 | 0.103 | 0.491 | -**0.709*** |
| Verrucomicrobia | Verrucomicrobiae | Verrucomicrobiales | 4.10% | 0.224 | 0.333 | -0.127 | -0.479 | **0.709*** | -0.152 | -0.188 | -0.297 | 0.236 |

*) p≥ 0.05
