# Supplementary material for: Changes in Cecal Microbiota and Mucosal Gene Expression Revealed New Aspects of Epizootic Rabbit Enteropathy
Source: PLoS One. 2014 Aug 22;9(8):e105707. doi: 10.1371/journal.pone.0105707 (PMC4141808; doi:10.1371/journal.pone.0105707)
Supplement: Table S11 — Correlation coefficients of the frequency of OTUs (frequency >0.1%) and gene expression in the ERE group. (DOCX) [file pone.0105707.s012.docx]

**Table S11**.- Correlation coefficients of the frequency of OTUs (frequency >0.1%) and gene expression in the ERE group.

| **PHYLUM** | **CLASS** | **ORDER** | **FAMILY** | **Freq** | **MUC1** | **MUC13** | **IL2** | **IFN-γ** | **MUC4** | **IL8** | **TNF-α** | **IL6** | **SPDEF** |
| --- | --- | --- | --- | --- | --- | --- | --- | --- | --- | --- | --- | --- | --- |
| Bacteroidetes | Bacteroidia | Bacteroidales | Rikenellaceae | 5.50% | -0.224 | -**0.673*** | 0.079 | 0.552 | -0.091 | 0.442 | 0.515 | 0.406 | -0.442 |
|  |  |  | Odoribacteraceae | 0.10% | -**0.721*** | -**0.842*** | **0**.**661*** | **0.685*** | -0.200 | 0.588 | 0.455 | **0.758*** | -**0.794*** |
| Firmicutes | Clostridia | Clostridiales | Catabacteriaceae | 4.50% | **0.709*** | 0.576 | -0.515 | -0.430 | 0.212 | -0.236 | -0.345 | -0.382 | 0.370 |
|  |  |  | Ruminococcaceae | 37.80% | -**0.721*** | -0.418 | **0.661*** | 0.576 | -0.127 | 0.321 | 0.333 | 0.455 | -0.176 |
| Proteobacteria | ε-Proteobacteria | Campylobacterales | Campylobacteraceae | 0.30% | -0.261 | -0.418 | 0.467 | 0.212 | 0.139 | 0.345 | 0.103 | 0.491 | -**0.709*** |
| Verrucomicrobia | Verrucomicrobiae | Verrucomicrobiales | Verrucomicrobiaceae | 4.10% | 0.224 | 0.333 | -0.127 | -0.479 | **0.709*** | -0.152 | -0.188 | -0.297 | 0.236 |

*) p≥ 0.05
