# Supplementary material for: Changes in Cecal Microbiota and Mucosal Gene Expression Revealed New Aspects of Epizootic Rabbit Enteropathy
Source: PLoS One. 2014 Aug 22;9(8):e105707. doi: 10.1371/journal.pone.0105707 (PMC4141808; doi:10.1371/journal.pone.0105707)
Supplement: Table S12 — Correlation coefficients in the ERE group of frequency of reads per Genera with gene expression (Ct relative to control) (counts from SILVA and correl coef Pearson and bilateral significance levels). Correlation coefficient in the ERE group: Orders Correlation Indices found in ERE rabbits between gene expression data (Ct) and OTUs. Data with a total frequency at least of 0.1%. (DOCX) [file pone.0105707.s013.docx]

**Table S12**.- Correlation coefficients in the ERE group of frequency of reads per Genera with gene expression (C_t_ relative to control) (counts from SILVA and correl coef Pearson and bilateral significance levels) Correlation coefficient in the ERE group: Orders Correlation Indices found in ERE rabbits between gene expression data (Ct) and OTUs. Data with a total frequency at least of 0.1%

|  |  | **MUC1** | | **MUC13** | | **IL2** | | **IFNG** | | **MUC4** | | **IL8** | | **TNF** | | **IL6** | | **SPDEF** | |
| --- | --- | --- | --- | --- | --- | --- | --- | --- | --- | --- | --- | --- | --- | --- | --- | --- | --- | --- | --- |
| **Genus** | Freq % |  | Sig. |  | Sig |  | Sig. |  | Sig. |  | Sig. |  | Sig. |  | Sig. |  | Sig. |  | Sig. |
| *Bacteroides* | 12,54 | 0,138 | 0,704 | -0,370 | 0,293 | 0,105 | 0,774 | 0,467 | 0,174 | 0,304 | 0,393 | 0,527 | 0,117 | **0,644*** | **0,044** | 0,544 | 0,104 | -0,517 | 0,126 |
| *Akkermansia* | 8,43 | 0,203 | 0,575 | 0,522 | 0,122 | -0,139 | 0,703 | -0,402 | 0,249 | **0,682*** | **0,030** | -0,269 | 0,452 | -0,116 | 0,750 | -0,354 | 0,316 | 0,367 | 0,297 |
| *Rikenella* | 3,42 | -0,222 | 0,537 | -0,535 | 0,111 | 0,154 | 0,671 | **0,696*** | **0,025** | -0,175 | 0,628 | 0,037 | 0,919 | 0,403 | 0,248 | 0,183 | 0,614 | -0,265 | 0,459 |
| *Alistipes* | 2,57 | -0,315 | 0,376 | **-0,735*** | **0,016** | 0,487 | 0,153 | **0,676*** | **0,032** | 0,437 | 0,207 | 0,365 | 0,300 | **0,653*** | **0,040** | 0,453 | 0,189 | -0,441 | 0,202 |
| *Lysinibacillus* | 1,00 | 0,123 | 0,735 | -0,225 | 0,532 | 0,176 | 0,626 | **0,714*** | **0,020** | -0,038 | 0,916 | 0,200 | 0,580 | 0,246 | 0,493 | 0,381 | 0,277 | -0,466 | 0,174 |
| *Thalassospira* | 0,71 | -0,336 | 0,343 | -0,358 | 0,309 | 0,504 | 0,137 | **0,679*** | **0,031** | -0,202 | 0,575 | -0,048 | 0,896 | 0,100 | 0,784 | 0,158 | 0,663 | -0,519 | 0,124 |
| *Clostridium* | 0,63 | 0,205 | 0,570 | -0,080 | 0,826 | -0,011 | 0,977 | 0,134 | 0,711 | **-0,701*** | **0,024** | 0,078 | 0,831 | -0,236 | 0,511 | 0,169 | 0,641 | -0,618 | 0,057 |
| *Campylobacter* | 0,55 | -0,207 | 0,565 | -0,109 | 0,764 | **0,667*** | **0,035** | 0,228 | 0,526 | -0,091 | 0,803 | -0,024 | 0,948 | -0,106 | 0,770 | 0,083 | 0,820 | -0,414 | 0,234 |
| *Odoribacter* | 0,16 | -0,413 | 0,235 | -0,425 | 0,221 | 0,338 | 0,340 | **0,673*** | **0,033** | -0,403 | 0,248 | -0,013 | 0,971 | 0,122 | 0,738 | 0,178 | 0,622 | -0,507 | 0,134 |
| *Butyricimonas* | 0,11 | -0,417 | 0,231 | -0,531 | 0,114 | **0,696*** | **0,025** | 0,280 | 0,433 | 0,612 | 0,060 | 0,123 | 0,736 | 0,392 | 0,263 | 0,150 | 0,678 | -0,296 | 0,406 |

*) p≥ 0.05
