# Supplementary material for: Changes in Cecal Microbiota and Mucosal Gene Expression Revealed New Aspects of Epizootic Rabbit Enteropathy
Source: PLoS One. 2014 Aug 22;9(8):e105707. doi: 10.1371/journal.pone.0105707 (PMC4141808; doi:10.1371/journal.pone.0105707)
Supplement: Table S13 — Identification of sequences from Group 5 described in Abecia et al. 2005 [21] . (DOCX) [file pone.0105707.s014.docx]

**Table S13**.- Identification of sequences from Group 5 described in Abecia et al. 2005 ([Abecia et al 2005](#_ENREF_1))

|  | Identification database | | |
| --- | --- | --- | --- |
| Accession Number | SILVA | RDP | Greengenes |
| AF275923 | \|  \| Bacteria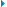Bacteroidetes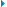Cytophagia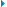Cytophagales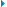Cytophagaceae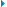uncultured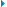 \| \| --- \| --- \| | \|  \| Bacteria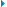"Bacteroidetes"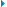"Sphingobacteria"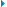"Sphingobacteriales"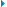Cytophagaceae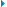Meniscus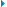 \| \| --- \| --- \| | \|  \| k__Bacteria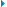p__Bacteroidetes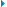c__Bacteroidia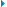o__Bacteroidales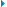Unclassified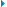 \| \| --- \| --- \| |
| AB062835 | \|  \| Bacteria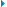Bacteroidetes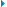Bacteroidia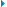Bacteroidales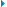Porphyromonadaceae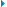uncultured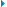 \| \| --- \| --- \| | Bacteria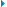"Bacteroidetes"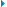"Bacteroidia"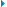"Bacteroidales"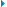"Porphyromonadaceae"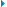Proteiniphilum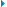 | \|  \| k__Bacteria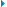p__Bacteroidetes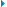c__Bacteroidia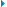o__Bacteroidales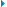f__Porphyromonadaceae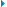g__Candidatus Azobacteroides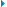Unclassified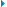 \| \| --- \| --- \| |
| AF376233 | \|  \| Bacteria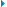Bacteroidetes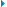Bacteroidia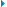Bacteroidales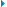Porphyromonadaceae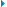Barnesiella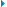 \| \| --- \| --- \| | \|  \| Bacteria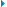"Bacteroidetes"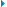"Bacteroidia"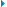"Bacteroidales"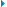"Porphyromonadaceae"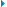Barnesiella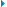 \| \| --- \| --- \| | k__Bacteria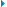p__Bacteroidetes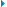c__Bacteroidia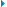o__Bacteroidales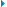Unclassified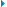 |
| AY244939 | \|  \| Bacteria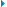Bacteroidetes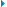Bacteroidia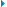Bacteroidales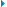RF16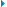 \| \| --- \| --- \| | \|  \| Bacteria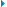"Bacteroidetes"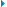"Bacteroidia"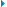"Bacteroidales"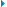"Porphyromonadaceae"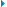unclassified_"Porphyromonadaceae" \| \| --- \| --- \| | \|  \| Unclassified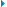 \| \| --- \| --- \| |
| AF371914 | \|  \| Bacteria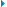Bacteroidetes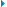Bacteroidia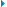Bacteroidales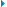RF16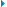 \| \| --- \| --- \| | \|  \| Bacteria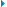"Bacteroidetes"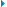"Bacteroidia"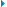"Bacteroidales"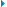"Porphyromonadaceae"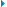Paludibacter \| \| --- \| --- \| | k__Bacteria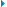p__Bacteroidetes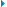c__Bacteroidia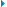o__Bacteroidales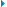f__Porphyromonadaceae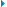Unclassified |
| AB009202 | \|  \| Bacteria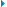Bacteroidetes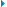Bacteroidia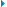Bacteroidales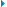RF16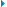 \| \| --- \| --- \| | \|  \| Bacteria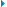"Bacteroidetes"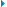"Bacteroidia"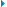"Bacteroidales"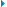"Porphyromonadaceae"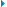Paludibacter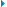 \| \| --- \| --- \| | \|  \| k__Bacteria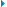p__Bacteroidetes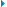c__Bacteroidia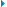o__Bacteroidales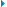f__Porphyromonadaceae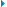g__Paludibacter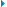 \| \| --- \| --- \| |

**REFERENCES**

Abecia L, Fondevila M, Balcells J, Edwards JE, Newbold CJ, McEwan NR (2005). Molecular profiling of bacterial species in the rabbit caecum. *FEMS Microbiol Lett* **244:** 111-115.
