# Supplementary material for: Changes in Cecal Microbiota and Mucosal Gene Expression Revealed New Aspects of Epizootic Rabbit Enteropathy
Source: PLoS One. 2014 Aug 22;9(8):e105707. doi: 10.1371/journal.pone.0105707 (PMC4141808; doi:10.1371/journal.pone.0105707)
Supplement: Table S14 — List of oligonucleotides used in this study for the quantification of gene expression through q-RT-PCR in rabbit's cecal mucosa. (DOCX) [file pone.0105707.s015.docx]

**Table S14.-** List of oligonucleotides used in this study for the quantification of gene expression through q-RT-PCR in rabbit’s cecal mucosa

| **Gene name** | **Accesion No.** | **Forward primer (5’ – 3’)** | **Reverse primer (5’ – 3’)** | **Amplicon size (bp)** |
| --- | --- | --- | --- | --- |
| ACTB | [NM_001101683.1](http://www.ncbi.nlm.nih.gov/entrez/viewer.fcgi?db=nucleotide&id=156119363) | TGGCGGGACACCCTCTCTCG | TGGGGATGCTCGCTCCAACG | 184 |
| GAPDH | [NM_001082253.1](http://www.ncbi.nlm.nih.gov/nuccore/NM_001082253.1) | GCCGCTTCTTCTCGTGCAG | ATGGATCATTGATGGCGACAACAT | 144 |
| MUC1 | XM_002715331.2 | CGTGTGTCAGTGCCGTCGCA | TACCCCCGTTGCCCGCAGAT | 170 |
| MUC13 | [XM_002716418.1](http://www.ncbi.nlm.nih.gov/entrez/viewer.fcgi?db=nucleotide&id=291400516) | GCACCTGTCTGCCGGGCTAC | CAGCAGATCACCGCGACCAC | 166 |
| MUC4 | LOC100350577 | CAAACATCTGCCACCACCTCCAG | GGTGAGAGGTAGACTTGTCCGCTG | 112 |
| IFN-G | NM_001081991.1 | TCTTACGGCTGTTACTGCCAGGAC | AGGGGTCCACCATTTGCCACATC | 101 |
| IL2 | [NM_001171099.1](http://www.ncbi.nlm.nih.gov/entrez/viewer.fcgi?db=nucleotide&id=283806642) | GCATTGCACTAACTCTTGCACTCCT | TCCAGCAGTAGCTGATCCAGTTGT | 94 |
| IL4 | [NM_001171106.1](http://www.ncbi.nlm.nih.gov/entrez/viewer.fcgi?db=nucleotide&id=283806659) | GGAACCTCTGCAGCATGGCGA | TGCATGGCGGTCTTTAGCCTGT | 100 |
| IL6 | NM_001082064.1 | TTCGGGGCTGATGGAGTTCCG | GCAACGGCTGGCTTGAGGGT | 174 |
| IL8 | NM_001082293.1 | ACAGAGCTTCGATGCCAGTGC | TCCTTGGGGTCCAGGCAGAGT | 158 |
| TNF- | [NM_001082263.1](http://www.ncbi.nlm.nih.gov/entrez/viewer.fcgi?db=nucleotide&id=126722915) | GCCACCACGCTCTTCTGCCT | GAGCTGGCCCTCCACTTGCG | 183 |
| SPDEF | [XM_002714628.1](http://www.ncbi.nlm.nih.gov/nuccore/XM_002714628.1) | CCGGTCATCGACAGTCAAGCC | CTGGTGCTCGGTCCACAGGAG | 216 |
